# Causal association of polyunsaturated fatty acids with chronic pain: a two-sample Mendelian randomization study

**DOI:** 10.3389/fnut.2023.1265928

**Published:** 2023-09-07

**Authors:** Yuxuan Dai, Yu Chen, Rui Gu, Chao Zhang, Rui Jiang

**Affiliations:** ^1^Department of Plastic Surgery, The Third Bethune Hospital of Jilin University, Changchun, China; ^2^Department of Surgical Oncology, The First Affiliated Hospital, Zhejiang University School of Medicine, Hangzhou, China; ^3^Department of Orthopedics, The Third Bethune Hospital of Jilin University, Changchun, China; ^4^Department of Ophthalmology, The Second Hospital of Jilin University, Changchun, China

**Keywords:** chronic pain, polyunsaturated fatty acid, Mendelian randomization, causal inference, genetics

## Abstract

**Background:**

Observational studies have indicated an association between polyunsaturated fatty acids (PUFAs) and chronic pain, but the potential causal link remains controversial. Here, we aimed to investigate whether a causal relationship exists between the concentration of circulating PUFAs and chronic pain as well as the direction of this association.

**Methods:**

We collected statistical data from relevant genome-wide association studies to explore the causal link between four PUFAs, along with the ratio of omega-6 fatty acids (FAs) to omega-3 FAs (omega-6:3 ratio), and chronic pain in eight specific body parts. We used the inverse-variance weighting (IVW) method for two-sample Mendelian randomization (MR) analysis and conducted supplementary analyses using four other methods (MR-Egger, weighted median, weighted mode, and simple mode). To verify the robustness of the MR study, we performed multiple sensitivity analyses.

**Results:**

The results revealed a negative correlation between omega-3 FAs [IVW, OR 95% CI: 0.952 (0.914, 0.991), *p* = 0.017] and docosahexaenoic acid (DHA) [IVW, OR 95% CI: 0.935 (0.893, 0.978), *p* = 0.003] with abnormal and pelvic pain. Furthermore, a positive correlation was observed between the omega-6:3 ratio [IVW, OR 95% CI: 1.057 (1.014, 1.101), *p* = 0.009] with abdominal and pelvic pain. Additionally, we found a negative correlation between omega-3 FAs [IVW, OR 95% CI: 0.947 (0.902, 0.994), *p* = 0.028] and lower back pain or sciatica. However, no causal relationship was found between the concentration of circulating PUFAs and pain in other body parts, including the face, throat and chest, joints, limbs, lower back, and gynecological parts. The robustness of these MR results was verified through multi-validity and retention method analyses.

**Conclusion:**

Our analysis suggests that higher circulating concentrations of omega-3 FAs and DHA and a lower omega-6:3 ratio are associated with a reduced risk of abdominal and pelvic pain. Additionally, a higher concentration of circulating omega-3 FAs is linked to a reduced risk of lower back pain and/or sciatica. These findings have major implications for the targeted prevention and treatment of chronic pain using PUFAs.

## 1. Introduction

Chronic pain is defined as a pain sensation that persists or recurs for over 3 months (1). Over 30% of the global population currently suffers from chronic pain, making it a major public health issue (2). Unlike acute pain, chronic pain is long-lasting and can persist for years or even longer, imposing a substantial burden on individuals and the economy (3, 4). Therefore, identifying risk and protective factors for chronic pain is crucial for its prevention and treatment.

Polyunsaturated fatty acids (PUFAs) are essential fatty acids (FAs) that cannot be synthesized by the body and must be obtained through dietary intake (5). PUFAs are key components of cell membranes and play a crucial role in cell structure and function (6). Additionally, PUFAs are involved in regulating inflammatory response, antithrombotic processes, lipid metabolism, and other physiological functions (7–9). Observational studies have shown an association between PUFA intake and the occurrence and severity of chronic pain (10–13). Omega-3 FAs can reduce inflammatory markers in chondrocytes, exerting anti-inflammatory and analgesic effects, whereas omega-6 FAs can promote inflammation and induce pain (14). However, a five-year randomized controlled study conducted in the United States found that omega-3 FA supplementation did not improve knee pain caused by osteoarthritis (15). Therefore, the causal relationship between PUFAs and chronic pain remains unclear.

Mendelian randomization (MR) utilizes genetic variation as an instrumental variable to infer causal relationships between exposure and disease outcome (16). By following the Mendelian inheritance law of “random distribution of parental alleles to offspring” during gamete formation, genetic variation is not influenced by traditional confounding factors such as socioeconomic status, environmental exposure, and behavioral factors. Thus, MR overcomes the limitations of traditional epidemiological studies, which can be affected by confounding factors and reverse causality (17). In this study, we aimed to investigate the causal relationship and its directionality between circulating PUFA concentrations and pain in different body sites using a two-sample MR analysis with large-scale open-access genome-wide association study (GWAS) pooled data.

## 2. Materials and methods

### 2.1. Study design

**Figure 1:**
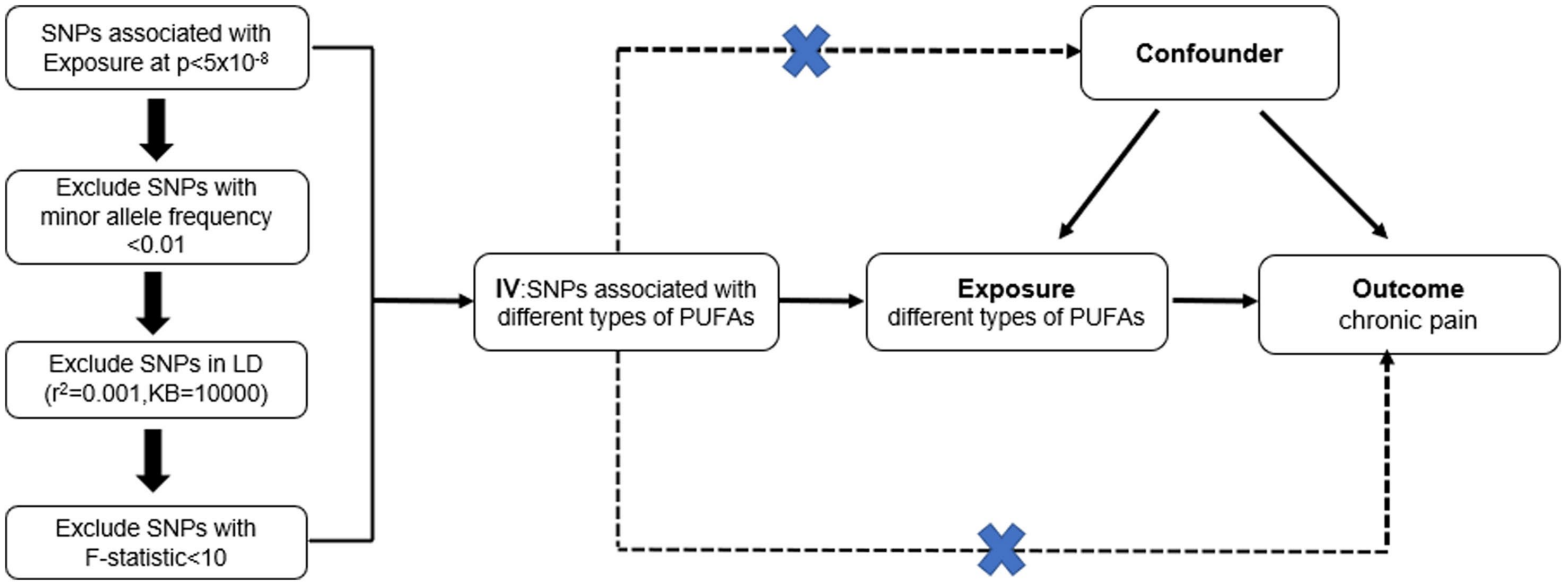
Mendelian randomized study design between different types of polyunsaturated fatty acid and chronic pain in different body parts.

In this study, MR was used to analyze the causal relationship between concentrations of circulating PUFAs, including omega-3 FAs, omega-6 FAs, linoleic acid, and docosahexaenoic acid (DHA), along with the ratio of omega-6 FAs to omega-3 FAs (omega-6:3 ratio), and pain in eight sites (Figure 1). The pain locations included the face, throat and chest, abdomen and pelvis, lower back and/or sciatica, joints, limbs, low back, and gynecological parts.

MR is a method used to infer a causal relationship between exposure factors and outcomes by analyzing the association between genetic variation and exposure factors, as well as genetic variation and outcomes. Therefore, MR studies require instrumental variables that meet three core assumptions: (1) strong and robust correlation between instrumental variables and exposure factors (association assumption); (2) independence of instrumental variables from confounding factors that affect the exposure–outcome relationship (independence hypothesis); (3) genetic variation affecting outcomes only through exposure factors and not through other pathways (exclusivity hypothesis) (18–20).

### 2.2. Data sources

All data from this study are publicly available GWAS summary statistics; therefore, no additional ethical approval or informed consent was required. Pooled data on circulating PUFA concentrations and pain in different sites were obtained from the IEU OPEN GWAS PROJECT database.^1^ To avoid the bias of population heterogeneity, only pooled data from European populations were used. Detailed information on the GWAS datasets is provided in Table 1.

**Table 1:** The list of Genome-wide summary association studies (GWAS) included in the Mendelian randomization study.

**Exposures**	**GWAS ID**	**Population**	**Year**	**Consortium**	**Participants**	**No. SNP**
Omega-3 FAs	met-d-Omega_3	European	2020	UK biobank	114,999	12,321,875
Omega-6 FAs	met-d-Omega_6	European	2020	UK biobank	114,999	12,321,875
Linoleic acid	met-d-LA	European	2020	UK biobank	114,999	12,321,875
DHA	met-d-DHA	European	2020	UK biobank	114,999	12,321,875
RO63	met-d-Omega_6_by_ Omega_3	European	2020	UK biobank	114,999	12,321,875

Outcome	GWAS ID	Population	Year	Consortium	Participants	No. SNP
Atypical facial pain	finn-b-G6_ATYFAC	European	2021	Finn Gen	701 cases/195,047 controls	16,380,408
Pain in throat and chest	finn-b-R18_PAIN_ THROAT_CHEST	European	2021	Finn Gen	24,609 cases/ 163,123 controls	16,380,345
Abdominal and pelvic pain	finn-b-R18_ABDOMI_ PELVIC_PAIN	European	2021	Finn Gen	49,416cases/ 161,968 controls	16,380,396
Lower back pain or/and sciatica	finn-b-M13_LOWBACK PAINORANDSCIATICA	European	2021	Finn Gen	19,509 cases/ 199,283 controls	16,380,466
Pain in joint	finn-b-JOINTPAIN	European	2021	Finn Gen	13,419 cases/ 131,550 controls	16,380,073
Pain in limb	finn-b-M13_LIMBPAIN	European	2021	Finn Gen	12,606 cases/ 167,641 controls	16,380,354
Low back pain	finn-b-M13_LOWBACK PAIN	European	2021	Finn Gen	13,178 cases/ 164,682 controls	16,380,287
Gynecological related pain	finn-b-N14_FEMGEN PAIN	European	2021	Finn Gen	3,316 cases/ 68,969 controls	16,376,838

FAs, fatty acids; DHA, Docosahexaenoic acid; No. SNP, number of SNPs included in the analysis; RO63, ratio of omega-6 fatty acids to omega-3 fatty acids; Gynecological related pain, pain and other conditions associated with female genital organs and menstrual cycle.

GWAS data on circulating PUFA concentrations were obtained from the UK biobank and included 114,999 European participants. The GWAS data for pain in eight body parts were obtained from the Finland database. The detailed sample sizes were as follows: atypical facial pain (701 cases and 195,047 controls), throat and chest pain (24,609 cases and 163,123 controls), abdominal and pelvic pain (49,416 cases and 161,968 controls), lower back pain or sciatica (19,509 cases and 199,283 controls), joint pain (13,419 cases and 131,550 controls), limb pain (12,606 cases and 167,641 controls), low back pain (13,178 cases and 164,682 controls), and gynecological-related pain (pain and other conditions associated with female genital organs and menstrual cycle; 3,316 cases and 68,969 controls). We performed an MR analysis using single nucleotide polymorphisms (SNPs) associated with circulating PUFA concentrations as exposure factors and pain in different body sites as outcome factors.

### 2.3. Selection of IVs

To fulfill the first MR hypothesis that SNPs must be strongly associated with circulating PUFA concentrations, SNPs significantly associated with circulating PUFA concentrations (*p* < 5 × 10^−8^, r^2^ < 0.001, genetic distance = 10,000 kb) were selected at the genome-wide level. Thereafter, to satisfy the second MR hypothesis that genetic variation is not associated with potential confounders, we queried the Phenoscanner database^2^ to ensure that the selected SNPs were not associated with known confounders. Finally, the F statistic was calculated to evaluate the presence of weak instrumental variable bias for the selected instrumental variables. *F* > 10 indicates the absence of weak instrumental variable bias, further supporting the association hypothesis. The calculation formula for F was as follows: *F* = [*R*^2^/(1 – *R*^2^)] × [(*N*-*K*-1)/*K*], where *N* is the sample size of exposure factors, *K* is the number of instrumental variables, and *R*^2^ is the proportion of variation of exposure factors explained by instrumental variables (21).

### 2.4. MR statistical analysis

We used two-sample MR to examine the causal relationship and its directionality between circulating PUFA concentrations and pain in different body sites. Inverse variance weighting (IVW) was used as the primary analysis method, whereas weighted median, MR-Egger, weighted mode, and simple mode were used as supplementary analysis methods. Cochrane Q value and MR-Egger intercept were used to assess heterogeneity and horizontal pleiotropy, respectively (22). The MR-Pleiotropy RESidual Sum and Outlier (MR-PRESSO) method was employed to detect and adjust for potential pleiotropy (23). Additionally, a leave-one-out analysis was conducted by deleting one SNP at a time to examine the influence of SNPs with potential horizontal pleiotropy on MR estimates (24). Sensitivity analyses were visually illustrated using scatter, funnel, and forest plots to demonstrate the robustness of the MR study. Odds ratios (ORs) and 95% confidence intervals (CIs) were used to present the causal effect between circulating PUFA concentrations and pain in different body sites. All statistical analyses were performed using R software (version 4.2.1) with the “TwoSampleMR” (25) and “MR-PRESSO” (26) packages. A significance level of *p* < 0.05 was considered to indicate a causal relationship.

## 3. Results

### 3.1. GWAS of PUFAs and pain in different body parts

We extracted 29–50 strongly associated SNPs from the GWAS dataset of circulating PUFA concentrations. The SNPs corresponding to different types of circulating PUFA concentrations are presented as specific features of IVs in Supplementary Tables 1–6. *F*-values were calculated for all SNPs with a value greater than 10.

### 3.2. Primary MR analysis

**Figure 2:**
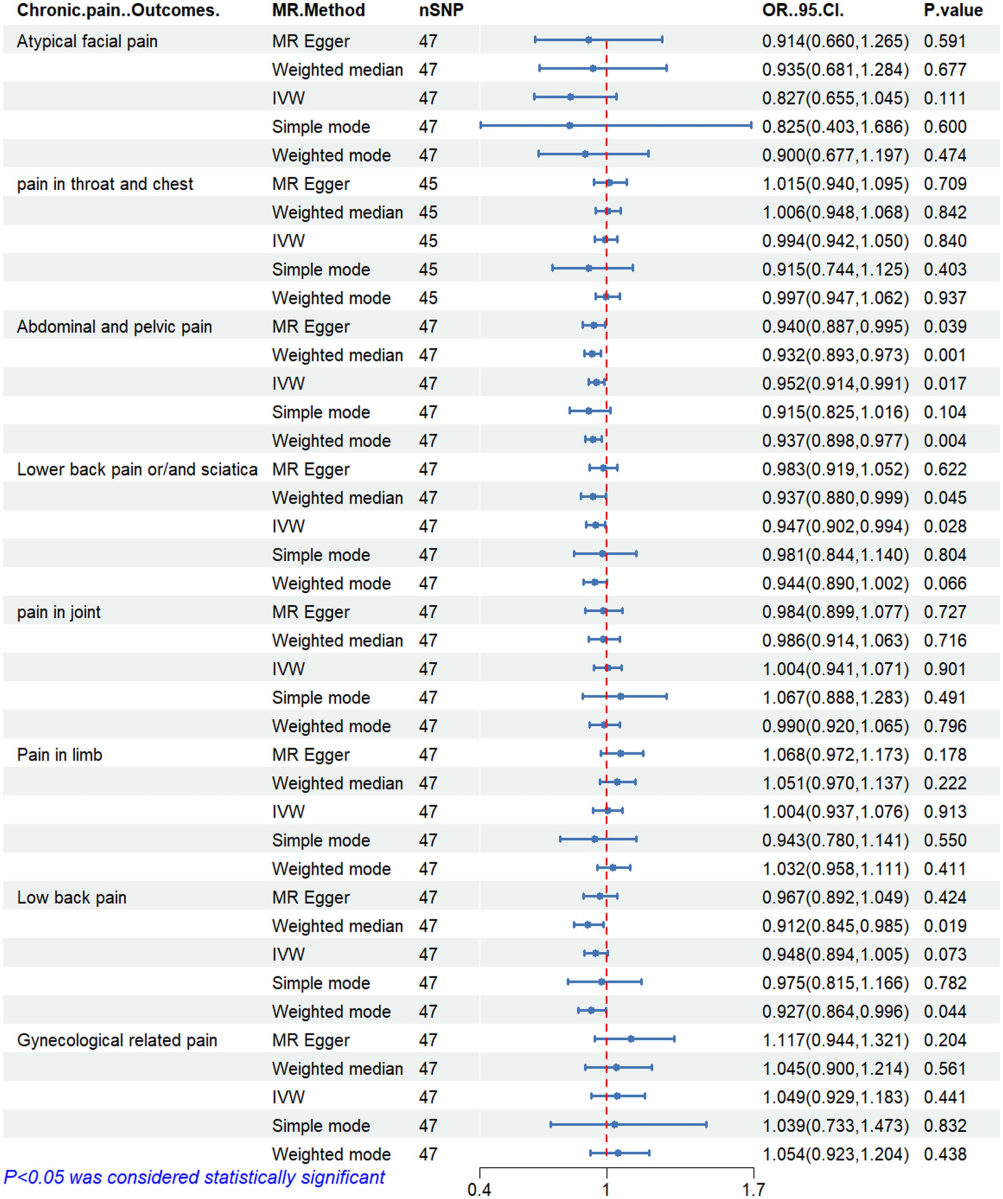
MR analysis results of Omega-3 fatty acids and pain in different body parts. No. SNP, number of SNPs included in the analysis; OR, Odds ratio; CI, confidence interval; IVW, Inverse-variance-weighted.

**Figure 3:**
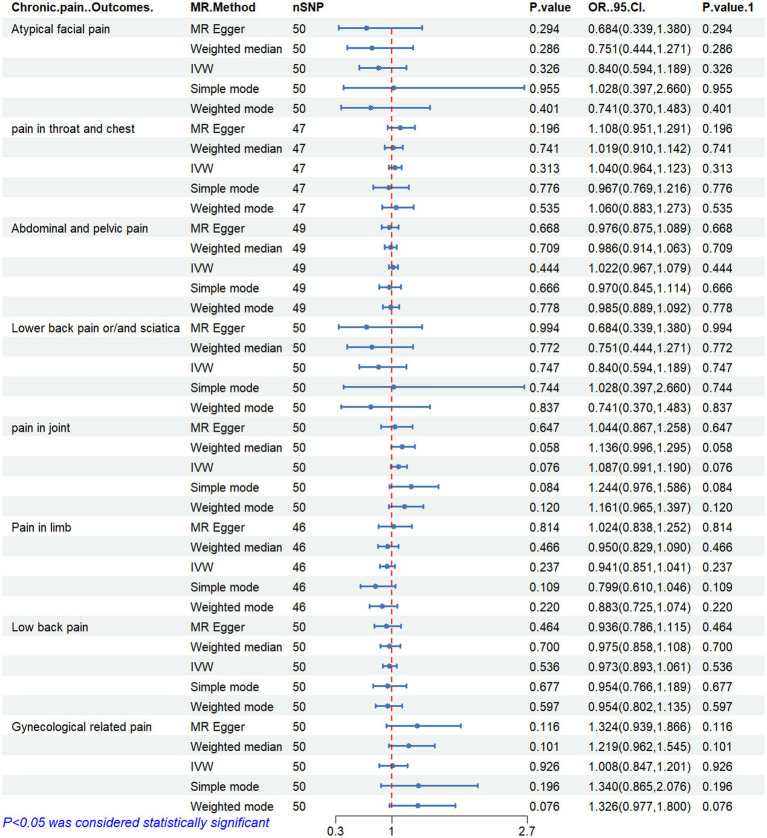
MR analysis results of Omega-6 fatty acids and pain in different body parts. No. SNP, number of SNPs included in the analysis; OR, Odds ratio; CI, confidence interval; IVW, Inverse-variance-weighted.

**Figure 4:**
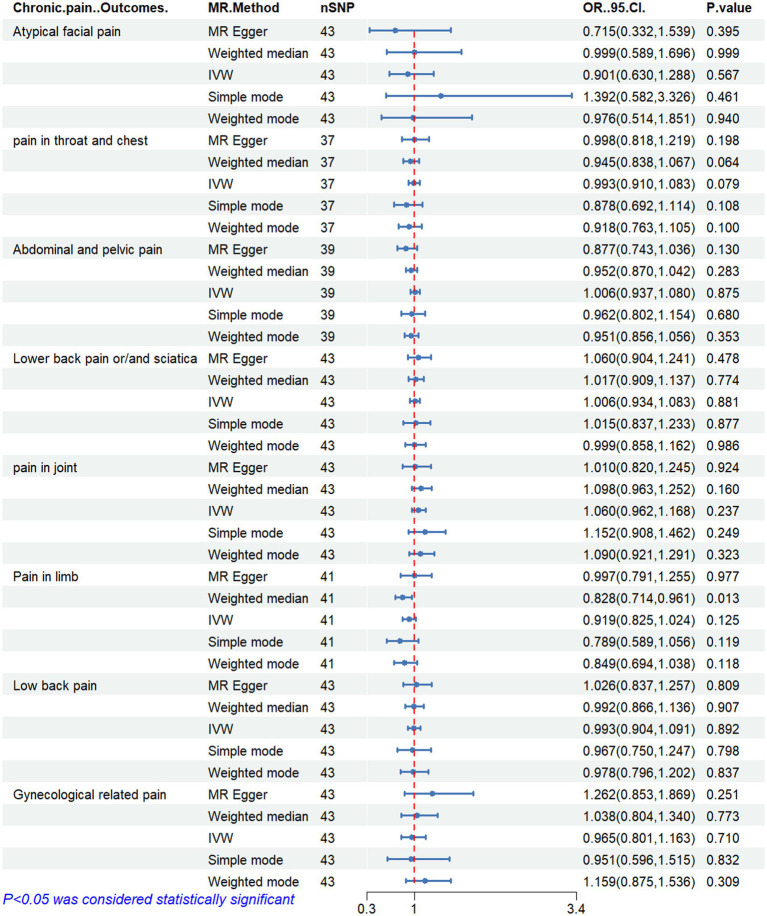
MR analysis results of linoleic acid and pain in different body parts. No. SNP, number of SNPs included in the analysis; OR, Odds ratio; CI, confidence interval; IVW, Inverse-variance-weighted.

**Figure 5:**
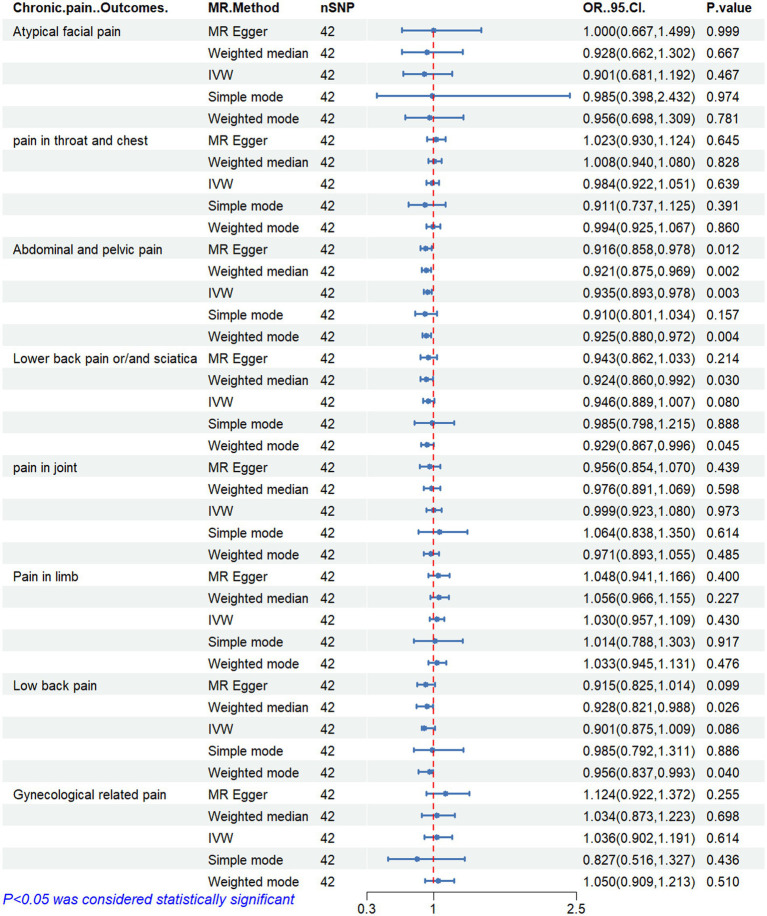
MR analysis results of docosahexaenoic acid and pain in different body parts. No. SNP, number of SNPs included in the analysis; OR, Odds ratio; CI, confidence interval; IVW, Inverse-variance-weighted.

**Figure 6:**
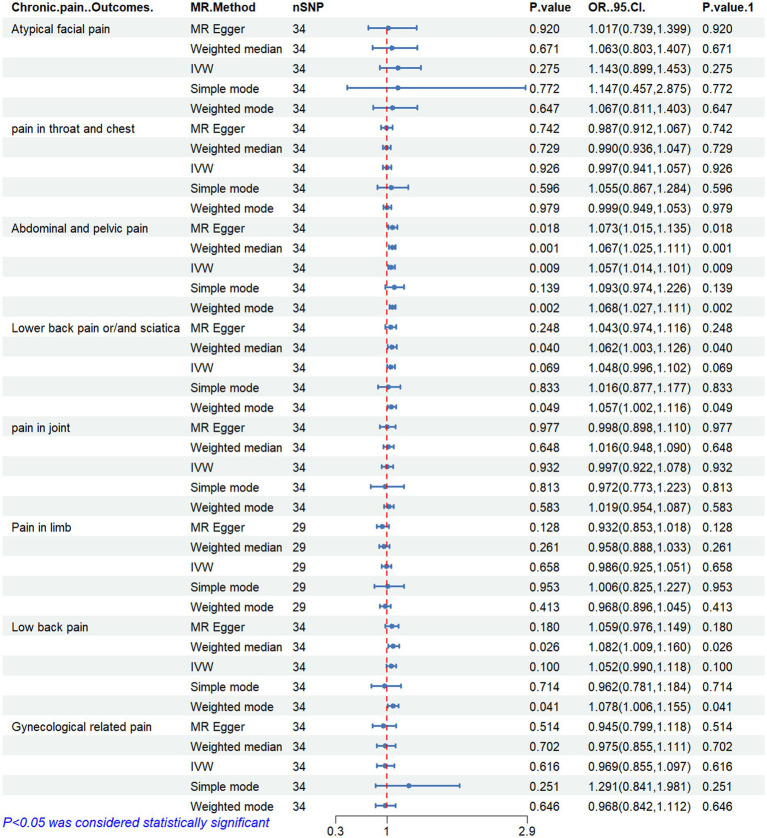
MR analysis results of ratio of omega-6 fatty acids to omega-3 fatty acids and pain in different body parts. No. SNP, number of SNPs included in the analysis; OR, Odds ratio; CI, confidence interval; IVW, Inverse-variance-weighted.

Figures 2–6 display the results of MR analysis examining the association between circulating PUFA concentrations and pain in different anatomical sites. In this MR study, we observed a negative correlation between omega-3 FAs [IVW, OR 95% CI: 0.952 (0.914, 0.991), *p* = 0.017] and DHA [IVW, OR 95% CI: 0.935 (0.893, 0.978), *p* = 0.003] with abdominal and pelvic pain. In contrast, the omega-6:3 ratio [IVW, OR 95% CI: 1.057 (1.014, 1.101), *p* = 0.009] showed a positive correlation with abdominal and pelvic pain. Additionally, we found that omega-3 FAs [IVW, OR 95% CI: 0.947 (0.902, 0.994), *p* = 0.028] were negatively correlated with lower back pain and/or sciatica. However, no causal link was observed between circulating PUFA concentrations and pain in other body sites, as measured using IVW (*p* > 0.05). Scatter plots (Supplementary Figures 1–5) were used to visualize the effect size for each MR analysis.

### 3.3. Supplementary and sensitivity analyses

In this MR study, we used additional analysis methods, namely weighted median, MR-Egger, weighted mode, and simple mode, as supplementary analyses to complement the primary IVW method. The Q and *p* values of MR-Egger and IVW analyses were used to assess heterogeneity, while the MR-Egger intercept and its *p*-value were used to detect pleiotropy. In cases where heterogeneity was present, causality was determined using a random effects model. The MR-PRESSO analysis was used to reduce heterogeneity and pleiotropy in the estimates of causal effects by removing outliers and reassessing causality. Notably, the MR-Egger intercept method did not detect any potential horizontal pleiotropy (*p* > 0.05), indicating the robustness of the study results. The outcomes of the heterogeneity and pleiotropy analyses are presented in Table 2. Additionally, a leave-one-out sensitivity analysis was conducted to verify the effect of each SNP site on the overall causal relationship. The results demonstrated that systematically removing individual SNPs and repeating the MR analysis did not yield significant differences in the aforementioned causal relationships (Supplementary Figures 6–10). Funnel plots depict the balanced distribution of each SNP (Supplementary Figures 11–15), and forest plots display estimates of individual SNP results (Supplementary Figures 16–20).

**Table 2:** Sensitivity analysis of Mendelian randomization studies of polyunsaturated fatty acids and different body parts.

Traits (Outcome)	Fatty acids (Exposure)	Heterogeneity test		MR-Egger pleiotropy test		MR-PRESSO	*F* statistics
						*p*-value	
		*Q*-value	*p*-value	Intercept	*p*-value		
Atypical facial pain	Omega-3 FAs	42.665	0.613	−0.012	0.391	0.637	264.64
	Omega-6 FAs	50.003	0.433	0.013	0.511	0.385	123.56
	Linoleic Acid	41.797	0.480	0.015	0.507	0.475	129.93
	DHA RO63	40.887 30.124	0.476 0.611	−0.011 0.018	0.487 0.284	0.561 0.655	210.76336.64
Pain in throat and chest	Omega-3 FAs	60.663	**0.048**	−0.002	0.454	0.066	264.54
	Omega-6 FAs	52.953	0.224	−0.004	0.356	0.255	118.22
	Linoleic Acid	41.770	0.234	−0.0003	0.952	0.246	118.17
	DHA RO63	59.616 50.852	**0.030 0.024**	−0.004 0.002	0.277 0.692	0.051 0.085	210.89338.73
Abdominal and pelvic pain	Omega-3 FAs	66.439	**0.026**	0.002	0.538	0.05	267.12
	Omega-6 FAs	56.939	0.177	0.003	0.354	0.179	125.39
	Linoleic Acid	53.823	**0.046**	0.008	0.084	0.061	110.86
	DHA RO63	50.547 46.758	0.146 0.057	0.002–0.002	0.415 0.404	0.2 0.136	210.95338.81
Lower back pain or/and sciatica	Omega-3 FAs	37.939	0.795	−0.004	0.126	0.805	264.39
	Omega-6 FAs	47.124	0.549	−0.001	0.861	0.621	123.48
	Linoleic Acid	40.128	0.553	−0.003	0.469	0.682	129.86
	DHA RO63	46.656 33.791	0.251 0.429	0.0003 0.004	0.933 0.829	0.306 0.496	209.51336.41
Pain in joint	Omega-3 FAs	54.863	0.173	0.002	0.530	0.201	270.57
	Omega-6 FAs	54.434	0.275	0.002	0.637	0.366	125.38
	Linoleic Acid	47.913	0.245	0.003	0.611	0.366	131.66
	DHA RO63	50.414 54.827	0.149**0.009**	0.004–0.0003	0.297 0.959	0.208 0.06	212.76341.84
Pain in limb	Omega-3 FAs	63.067	**0.048**	−0.008	0.071	0.066	266.62
	Omega-6 FAs	57.535	0.100	−0.006	0.343	0.102	129.32
	Linoleic Acid	55.155	0.056	−0.005	0.441	0.058	133.56
	DHA RO63	44.484 28.356	0.327 0.446	−0.002 0.008	0.671 0.083	0.38 0.482	210.69363.87
Low back pain	Omega-3 FAs	43.202	0.590	−0.002	0.491	0.575	266.87
	Omega-6 FAs	49.298	0.461	0.002	0.620	0.519	124.25
	Linoleic Acid	46.868	0.280	−0.002	0.730	0.438	130.59
	DHA RO63	42.586 33.900	0.403 0.424	0.002 0.004	0.490 0.822	0.412 0.416	210.82338.60
Gynecological related pain	Omega-3 FAs	54.807	0.175	−0.008	0.297	0.234	288.78
	Omega-6 FAs	56.404	0.218	−0.018	0.080	0.146	130.77
	Linoleic Acid	51.101	0.158	−0.018	0.136	0.15	143.53
	DHA RO63	44.903 39.586	0.312 0.200	−0.009 0.009	0.271 0.666	0.382 0.311	222.02357.52

DHA, Docosahexaenoic acid; RO63, ratio of omega-6 FAs to omega-3 FAs; Gynecological related pain, pain and other conditions associated with female genital organs and menstrual cycle; *p* < 0.05 is set as the significant threshold. *p* < 0.05 was considered statistically significant.

## 4. Discussion

We used MR to systematically assess the causal relationship and its direction between four PUFAs and omega-6:3 ratios and pain in eight body parts. The results revealed a negative correlation between the concentration of omega-3 FAs and DHA and the occurrence of abdominal and pelvic pain. Conversely, a positive correlation was observed between the omega-6:3 ratio and abdominal and pelvic pain. Furthermore, we observed a negative association between omega-3 FA concentration and lower back pain and/or sciatica. However, no causal relationship was found between circulating PUFA concentrations and pain in other body sites.

Omega-3 fatty acids have been found to have anti-inflammatory and analgesic effects, while Omega-6 fatty acids promote inflammation (27). Research has demonstrated that omega-3 fatty acids can influence cellular signaling pathways by altering the composition and function of cell membranes, thus reducing inflammatory responses (28). Specifically, DHA, one of the Omega-3 fatty acids, can reduce inflammation by inhibiting the synthesis of the inflammatory mediator prostaglandin E2 and combating oxidative stress (29, 30). Additionally, studies have shown that higher ratios of Omega-6 to Omega-3 fatty acids are associated with an increased risk of cardiovascular disease, autoimmune diseases, and cancer (31, 32). Therefore, maintaining a proper balance of omega-3 and Omega-6 fatty acid intake is crucial for maintaining good health. In theory, this evidence supports that higher circulating concentrations of omega-3 FAs and DHA, along with lower levels of the omega-6 to omega-3 ratio, may reduce the risk of abdominal and pelvic pain.

Several observational studies have explored the association between PUFAs and chronic pain (33–37). In a randomized controlled study, the infusion of omega-3 FAs demonstrated efficacy in alleviating pain caused by rheumatoid arthritis (38). Another study indicated that dietary supplementation with omega-3 FAs and DHA could reduce the incidence and progression of pain in the older population (39). However, omega-6 FAs may contribute to inflammation, as significantly elevated levels of omega-6 FA metabolites were reported in women with irritable bowel syndrome (40). Similarly, another study found a higher omega-6:3 ratio to be associated with orofacial pain, headache, and low back pain (41). In a randomized controlled study, joint pain in patients with inflammatory bowel disease was effectively ameliorated by correcting the omega-6:3 ratio (42). Our MR study produced consistent results, demonstrating that higher omega-6:3 ratios elevate the risk of abdominal pain, whereas higher circulating omega-3 FAs and DHA concentrations alleviate abdominal pain. However, no causal link was observed with joint pain.

Although observational studies have shown an association between PUFAs and chronic pain, only a few MR studies have been conducted. To the best of our knowledge, only one MR study has investigated the relationship between omega-3 FAs and low back pain risk, revealing that genetically increasing circulating omega-3 FA concentrations can reduce this risk (43). However, our findings do not support this conclusion. In our MR study, only the weighted median and weighted mode methods indicated a causal relationship between circulating omega-3 FA concentrations and low back pain, as measured using the IVW method [OR 95% CI: 0.948 (0.894, 1.005), *p* = 0.073], but no causal relationship was found between them. Notably, the sample size of our study was nearly twice that of the aforementioned MR study. Moreover, our study data was derived from different patient populations, sourced from the UK biobank and Finland genome project (Finn Gen), effectively avoiding sample duplication. Additionally, our findings remained robust across multiple analytical approaches, exhibiting no heterogeneity or pleiotropy. Although this MR study represents the largest and most comprehensive investigation to date in terms of sample size, FA types, and pain locations, future studies will require larger datasets from genomic association studies to obtain more accurate and reliable results.

This study possesses several advantages. First, this is the most comprehensive MR study to date exploring the relationship between PUFAs and pain. We included four different types of PUFAs and the omega-6:3 ratio while investigating causal associations with pain in eight body parts. This comprehensive approach enhances the coverage and credibility of our results. Second, compared with observational studies, MR studies overcome the effects of confounding variables and reverse causality. Third, we selected SNPs that satisfied the MR hypothesis as IVs, all of which exhibited strong associations with PUFAs. Lastly, all SNPs were derived from European populations, thereby minimizing the possibility of population stratification bias.

Nonetheless, this study has some limitations. First, it relied on publicly available pooled data from GWASs, which restricted our ability to analyze other subtypes of PUFAs (e.g., alpha-linolenic, eicosapentaenoic, and arachidonic acids) in investigating the relationship between PUFAs and pain. Second, despite the utilization of a large GWAS database, our study might have been unable to detect small causal relationships. Third, this study can only establish the relationship between genes and phenotypes but cannot elucidate the biological mechanism of these relationships. Therefore, the occurrence and development of complex phenotypes cannot be fully explained. Lastly, the study population primarily consisted of individuals of European ancestry, limiting the generalizability to other ethnic populations.

## 5. Conclusion

This MR study suggests that higher circulating omega-3 FA and DHA concentrations and lower omega-6:3 ratios are associated with a decreased risk of abdominal and pelvic pain. Maintaining an appropriate omega-6:3 ratio is crucial for the prevention and management of abdominal and pelvic pain. Furthermore, higher circulating omega-3 FA concentrations may reduce the risk of lower back pain and/or sciatica. However, no causal relationship was found between circulating PUFA concentrations and pain in other body parts, including pain related to the face, throat and chest, joints, limbs, low back pain, and gynecological sites. These findings contribute to the targeted prevention and treatment of chronic pain through the use of PUFAs.

## Data availability statement

The datasets presented in this study can be found in online repositories. The names of the repository/repositories and accession number (s) can be found in the article/Supplementary material.

## Author contributions

YD: Writing – review & editing, Conceptualization, Writing – original draft. YC: Conceptualization, Writing – review & editing, Data curation, Software. RG: Writing – review & editing, Supervision, Validation. RJ: Writing – review & editing, Project administration, Resources, Visualization. CZ: Data curation, Software, Writing – review & editing, Visualization.

## Funding

The author(s) declare that no financial support was received for the research, authorship, and/or publication of this article.

## Conflict of interest

The authors declare that the research was conducted in the absence of any commercial or financial relationships that could be construed as a potential conflict of interest.

## Publisher’s note

All claims expressed in this article are solely those of the authors and do not necessarily represent those of their affiliated organizations, or those of the publisher, the editors and the reviewers. Any product that may be evaluated in this article, or claim that may be made by its manufacturer, is not guaranteed or endorsed by the publisher.

## Footnotes

^1^https://gwas.mrcieu.ac.uk, accessed on June 10, 2023

^2^ http://www.phenoscanner.medschl.cam.ac.uk/
