# Supplementary material for: Causal association of polyunsaturated fatty acids with chronic pain: a two-sample Mendelian randomization study
Source: Front Nutr. 2023 Sep 7;10:1265928. doi: 10.3389/fnut.2023.1265928 (PMC10512421; doi:10.3389/fnut.2023.1265928)
Supplement: Supplementary file 1 [file Data_Sheet_1.ZIP › Supplementary figures and tables/Supplementary figure legends.docx]

**Supplementary Figure 1.** Scatter plots of causal estimates of exposure (Omega-3 fatty acids) on outcomes. A: Atypical facial pain; B: Pain in throat and chest; C: Abdominal and pelvic pain; D: Lower back pain or/and sciatica; E: Pain in joint; F: Pain in limb; G: Low back pain; H: Gynecological related pain.

**Supplementary Figure 2.** Scatter plots of causal estimates of exposure (Omega-6 fatty acids) on outcomes. A: Atypical facial pain; B: Pain in throat and chest; C: Abdominal and pelvic pain; D: Lower back pain or/and sciatica; E: Pain in joint; F: Pain in limb; G: Low back pain; H: Gynecological related pain.

**Supplementary Figure 3.** Scatter plots of causal estimates of exposure (Linoleic acid) on outcomes. A: Atypical facial pain; B: Pain in throat and chest; C: Abdominal and pelvic pain; D: Lower back pain or/and sciatica; E: Pain in joint; F: Pain in limb; G: Low back pain; H: Gynecological related pain.

**Supplementary Figure 4.** Scatter plots of causal estimates of exposure (Docosahexaenoic acid) on outcomes. A: Atypical facial pain; B: Pain in throat and chest; C: Abdominal and pelvic pain; D: Lower back pain or/and sciatica; E: Pain in joint; F: Pain in limb; G: Low back pain; H: Gynecological related pain.

**Supplementary Figure 5.** Scatter plots of causal estimates of exposure (Ratio of omega-6 fatty acids to omega-3 fatty acids) on outcomes. A: Atypical facial pain; B: Pain in throat and chest; C: Abdominal and pelvic pain; D: Lower back pain or/and sciatica; E: Pain in joint; F: Pain in limb; G: Low back pain; H: Gynecological related pain.

**Supplementary Figure 6.** Leave-one-out stability tests causal estimates of exposure (Omega-3 fatty acids) on outcomes. A: Atypical facial pain; B: Pain in throat and chest; C: Abdominal and pelvic pain; D: Lower back pain or/and sciatica; E: Pain in joint; F: Pain in limb; G: Low back pain; H: Gynecological related pain.

**Supplementary Figure 7.** Leave-one-out stability tests causal estimates of exposure (Omega-6 fatty acids) on outcomes. A: Atypical facial pain; B: Pain in throat and chest; C: Abdominal and pelvic pain; D: Lower back pain or/and sciatica; E: Pain in joint; F: Pain in limb; G: Low back pain; H: Gynecological related pain.

**Supplementary Figure 8.** Leave-one-out stability tests causal estimates of exposure (Linoleic acid) on outcomes. A: Atypical facial pain; B: Pain in throat and chest; C: Abdominal and pelvic pain; D: Lower back pain or/and sciatica; E: Pain in joint; F: Pain in limb; G: Low back pain; H: Gynecological related pain.

**Supplementary Figure 9.** Leave-one-out stability tests causal estimates of exposure (Docosahexaenoic acid) on outcomes. A: Atypical facial pain; B: Pain in throat and chest; C: Abdominal and pelvic pain; D: Lower back pain or/and sciatica; E: Pain in joint; F: Pain in limb; G: Low back pain; H: Gynecological related pain.

**Supplementary Figure 10.** Leave-one-out stability tests causal estimates of exposure (Ratio of omega-6 fatty acids to omega-3 fatty acids) on outcomes. A: Atypical facial pain; B: Pain in throat and chest; C: Abdominal and pelvic pain; D: Lower back pain or/and sciatica; E: Pain in joint; F: Pain in limb; G: Low back pain; H: Gynecological related pain.

**Supplementary Figure 11.** Funnel plots of causal estimates of exposure (Omega-3 fatty acids) on outcomes. A: Atypical facial pain; B: Pain in throat and chest; C: Abdominal and pelvic pain; D: Lower back pain or/and sciatica; E: Pain in joint; F: Pain in limb; G: Low back pain; H: Gynecological related pain.

**Supplementary Figure 12.** Funnel plots of causal estimates of exposure (Omega-6 fatty acids) on outcomes. A: Atypical facial pain; B: Pain in throat and chest; C: Abdominal and pelvic pain; D: Lower back pain or/and sciatica; E: Pain in joint; F: Pain in limb; G: Low back pain; H: Gynecological related pain.

**Supplementary Figure 13.** Funnel plots of causal estimates of exposure (Linoleic acid) on outcomes. A: Atypical facial pain; B: Pain in throat and chest; C: Abdominal and pelvic pain; D: Lower back pain or/and sciatica; E: Pain in joint; F: Pain in limb; G: Low back pain; H: Gynecological related pain.

**Supplementary Figure 14.** Funnel plots of causal estimates of exposure (Docosahexaenoic acid) on outcomes. A: Atypical facial pain; B: Pain in throat and chest; C: Abdominal and pelvic pain; D: Lower back pain or/and sciatica; E: Pain in joint; F: Pain in limb; G: Low back pain; H: Gynecological related pain.

**Supplementary Figure 15.** Funnel plots of causal estimates of exposure (Ratio of omega-6 fatty acids to omega-3 fatty acids) on outcomes. A: Atypical facial pain; B: Pain in throat and chest; C: Abdominal and pelvic pain; D: Lower back pain or/and sciatica; E: Pain in joint; F: Pain in limb; G: Low back pain; H: Gynecological related pain.

**Supplementary Figure 16.** Forest plots of causal estimates of exposure (Omega-3 fatty acids) on outcomes. A: Atypical facial pain; B: Pain in throat and chest; C: Abdominal and pelvic pain; D: Lower back pain or/and sciatica; E: Pain in joint; F: Pain in limb; G: Low back pain; H: Gynecological related pain.

**Supplementary Figure 17.** Forest plots of causal estimates of exposure (Omega-6 fatty acids) on outcomes. A: Atypical facial pain; B: Pain in throat and chest; C: Abdominal and pelvic pain; D: Lower back pain or/and sciatica; E: Pain in joint; F: Pain in limb; G: Low back pain; H: Gynecological related pain.

**Supplementary Figure 18.** Forest plots of causal estimates of exposure (Linoleic acid) on outcomes. A: Atypical facial pain; B: Pain in throat and chest; C: Abdominal and pelvic pain; D: Lower back pain or/and sciatica; E: Pain in joint; F: Pain in limb; G: Low back pain; H: Gynecological related pain.

**Supplementary Figure 19.** Forest plots of causal estimates of exposure (Docosahexaenoic acid) on outcomes. A: Atypical facial pain; B: Pain in throat and chest; C: Abdominal and pelvic pain; D: Lower back pain or/and sciatica; E: Pain in joint; F: Pain in limb; G: Low back pain; H: Gynecological related pain.

**Supplementary Figure 20.** Forest plots of causal estimates of exposure (Ratio of omega-6 fatty acids to omega-3 fatty acids) on outcomes. A: Atypical facial pain; B: Pain in throat and chest; C: Abdominal and pelvic pain; D: Lower back pain or/and sciatica; E: Pain in joint; F: Pain in limb; G: Low back pain; H: Gynecological related pain.
