# Supplementary figures and images for: Causal association of polyunsaturated fatty acids with chronic pain: a two-sample Mendelian randomization study
Source: Front Nutr. 2023 Sep 7;10:1265928. doi: 10.3389/fnut.2023.1265928 (PMC10512421; doi:10.3389/fnut.2023.1265928)

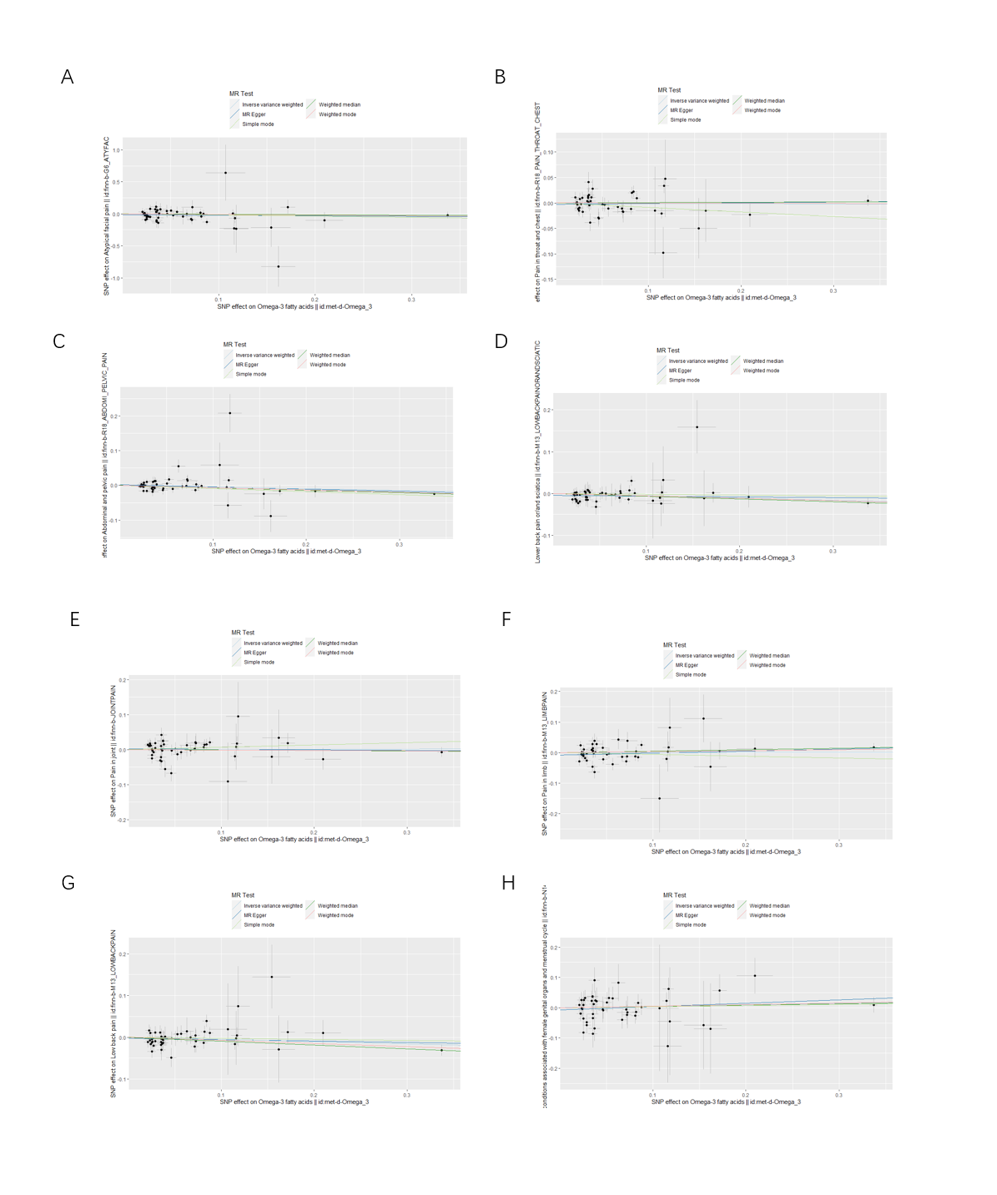

Supplement: Supplementary file 1 [file Data_Sheet_1.ZIP › Supplementary figures and tables/Supplementary Figure 1.TIFF]

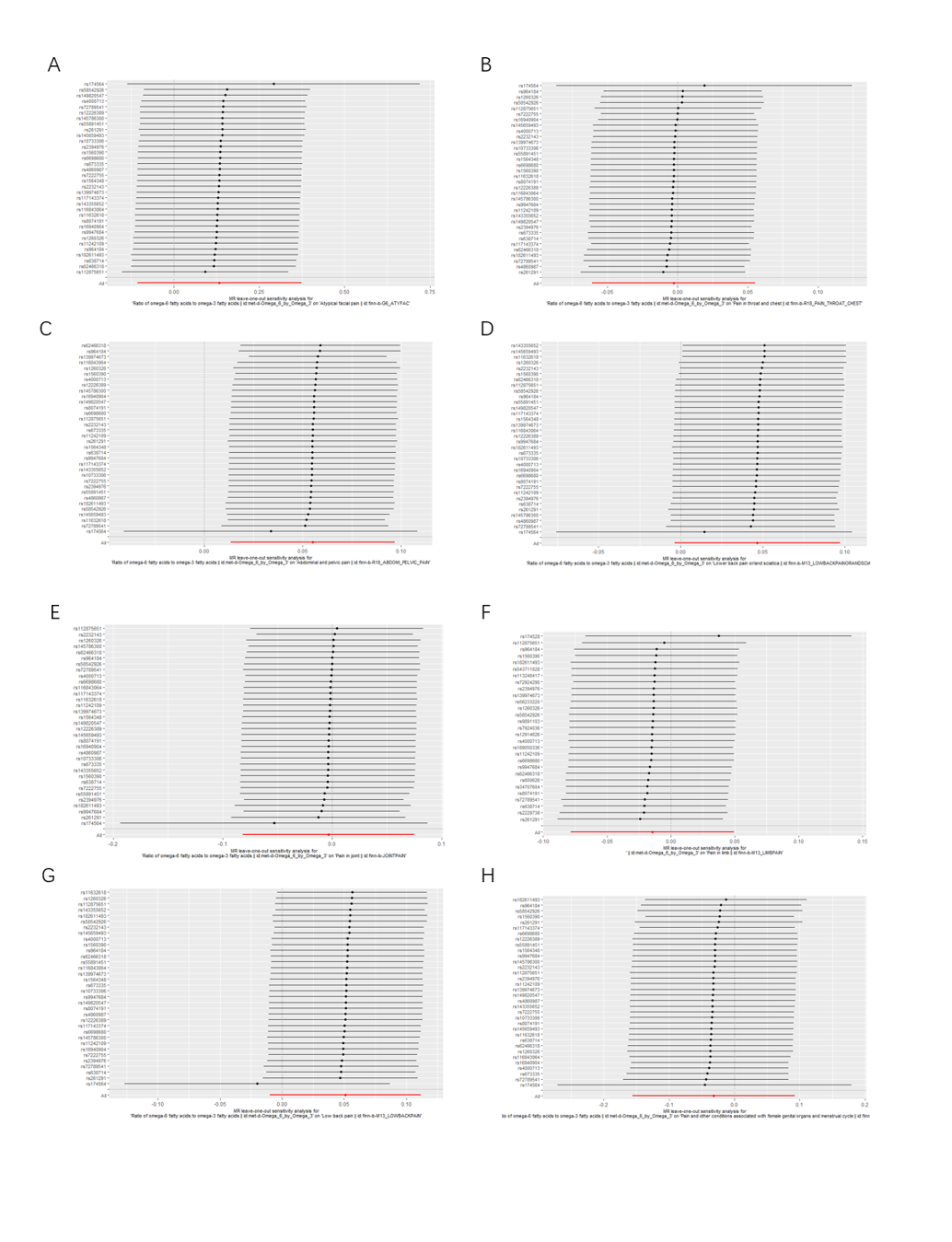

Supplement: Supplementary file 1 [file Data_Sheet_1.ZIP › Supplementary figures and tables/Supplementary Figure 10.TIFF]

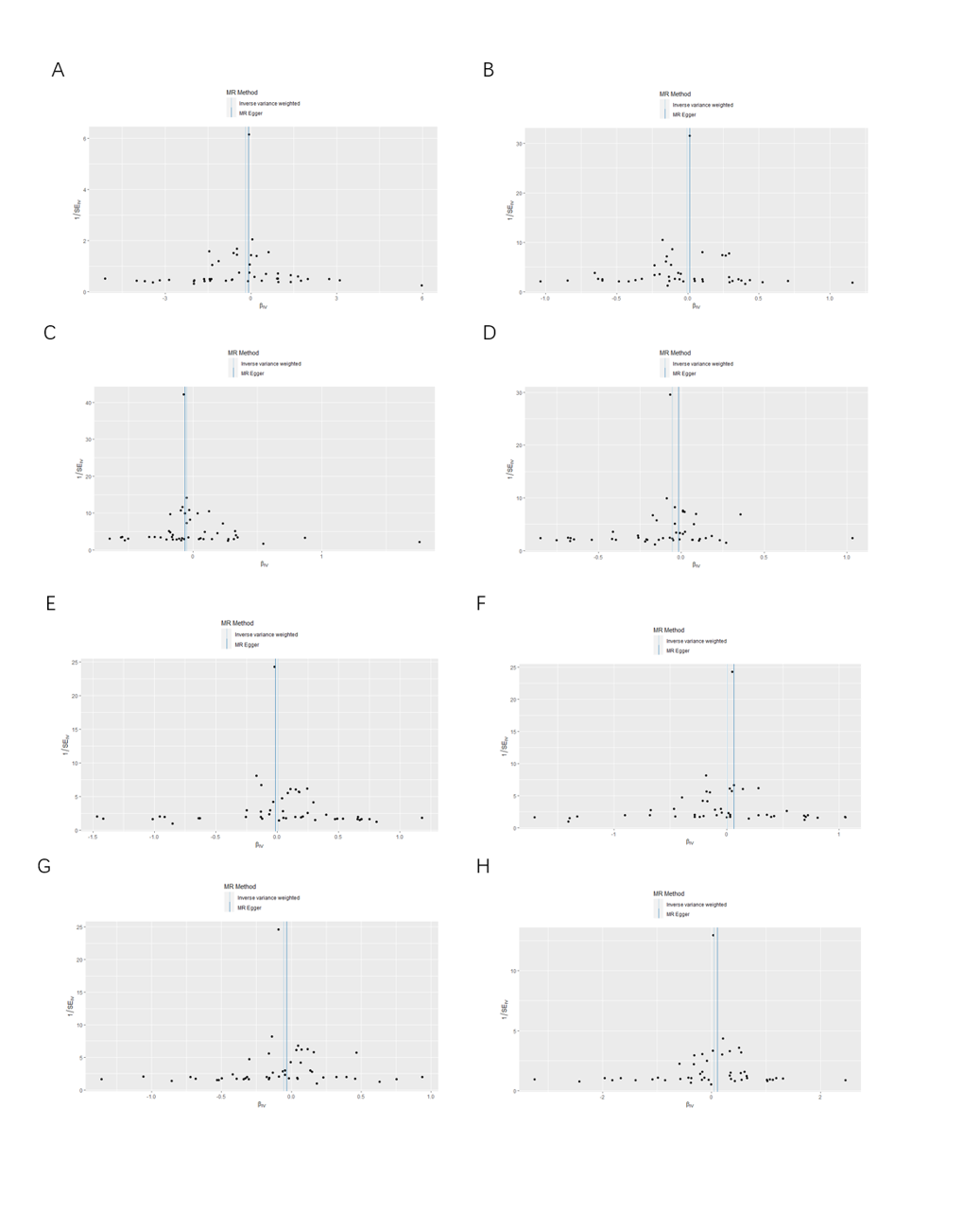

Supplement: Supplementary file 1 [file Data_Sheet_1.ZIP › Supplementary figures and tables/Supplementary Figure 11.TIFF]

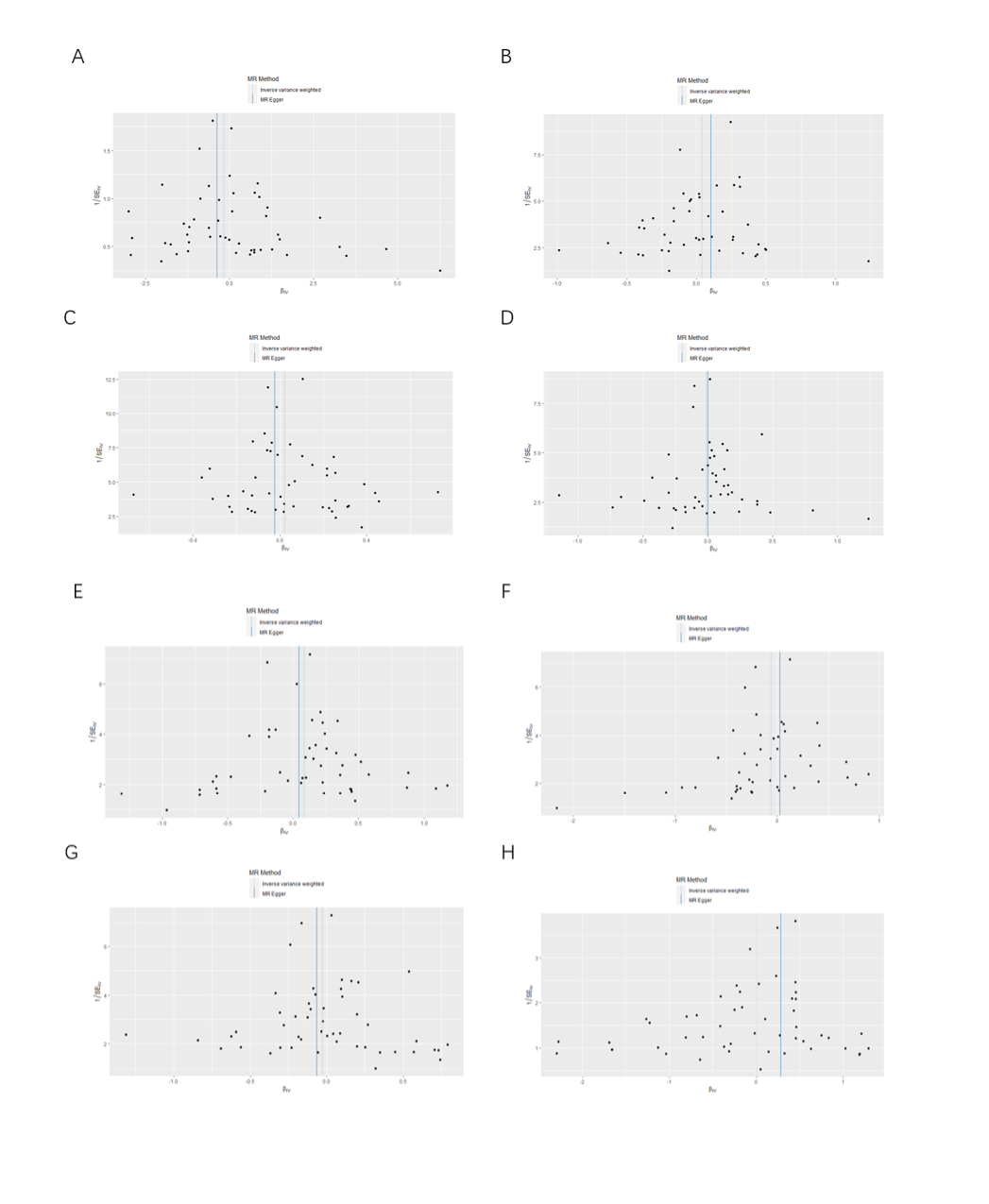

Supplement: Supplementary file 1 [file Data_Sheet_1.ZIP › Supplementary figures and tables/Supplementary Figure 12.TIFF]

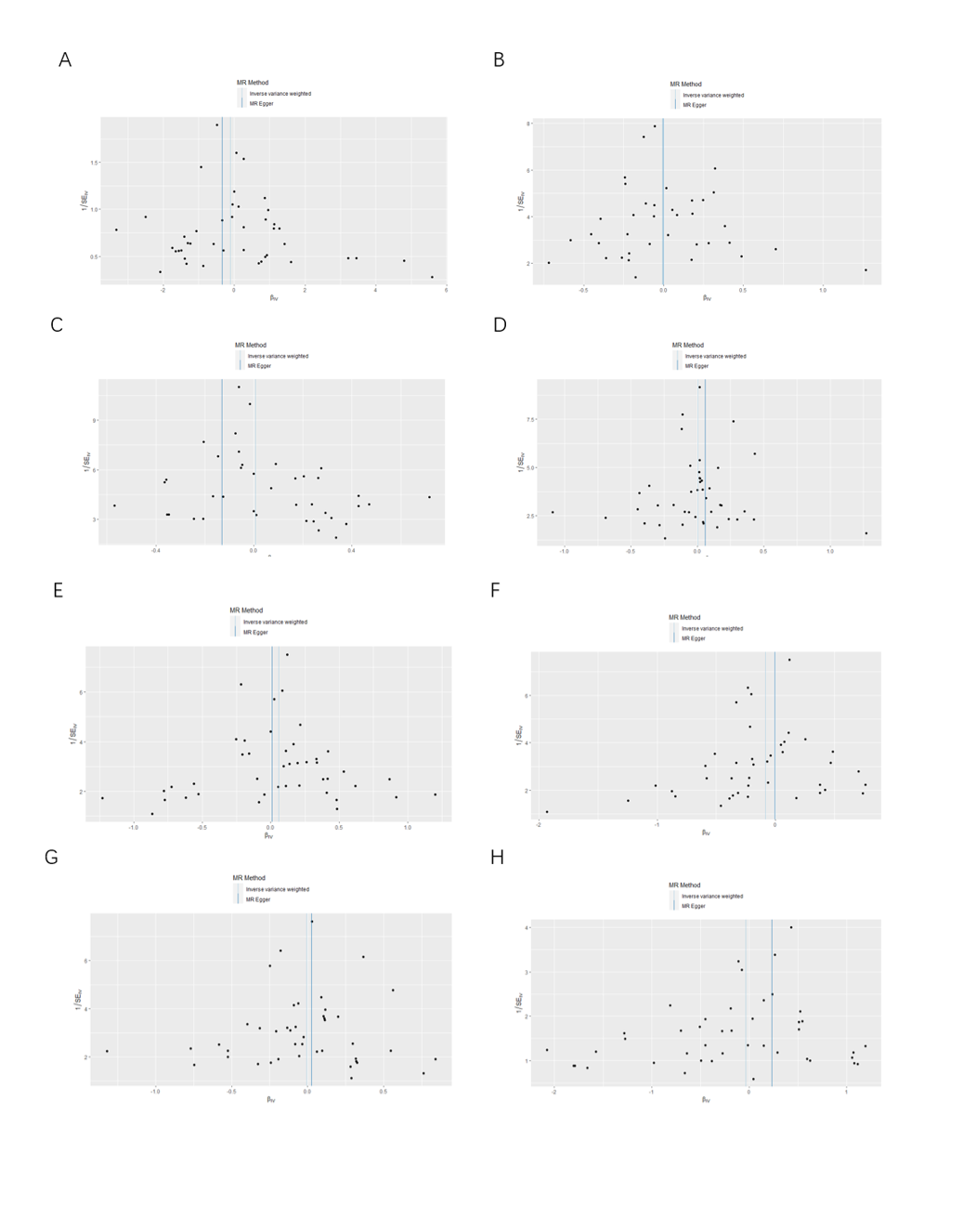

Supplement: Supplementary file 1 [file Data_Sheet_1.ZIP › Supplementary figures and tables/Supplementary Figure 13.TIFF]

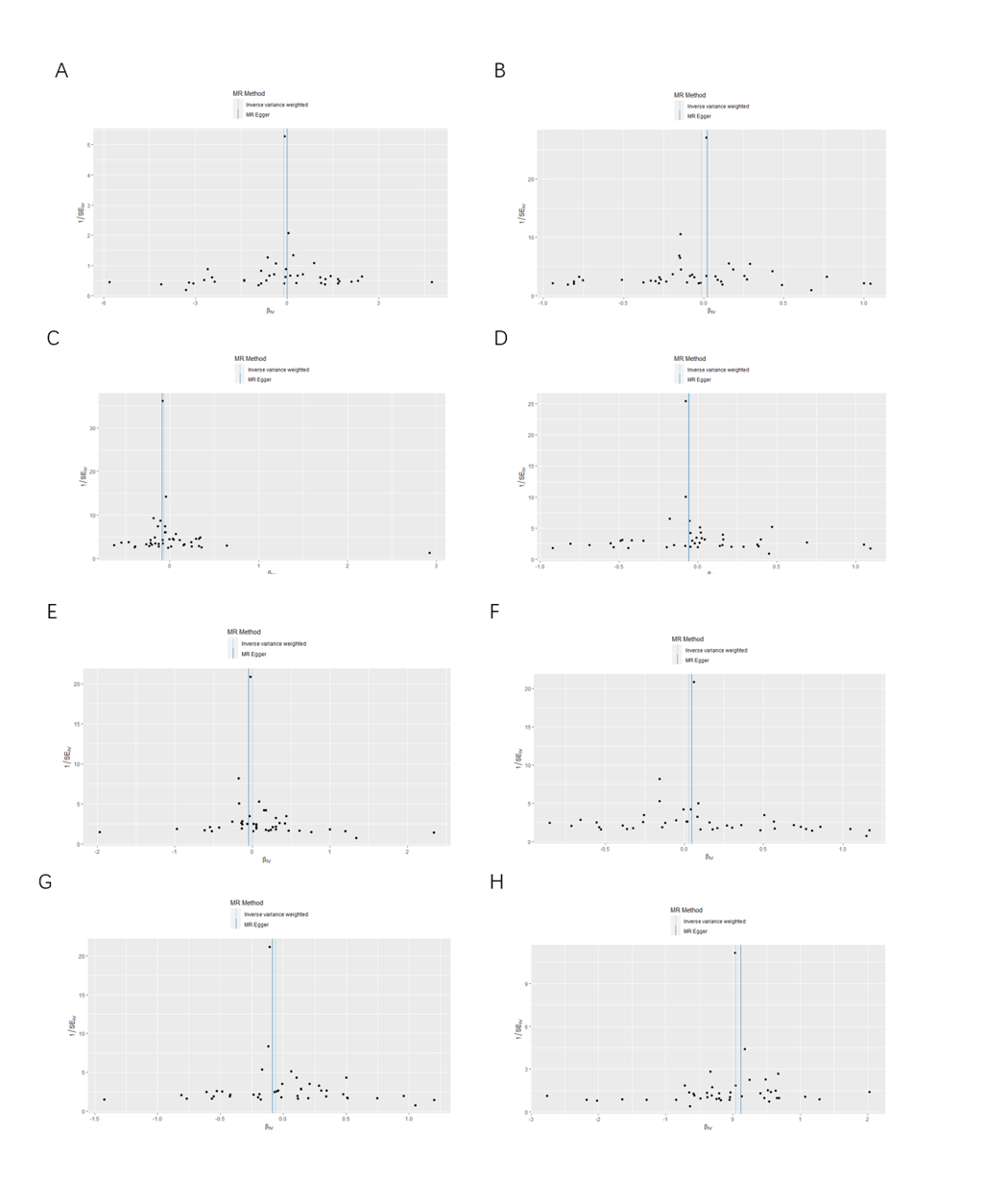

Supplement: Supplementary file 1 [file Data_Sheet_1.ZIP › Supplementary figures and tables/Supplementary Figure 14.TIFF]

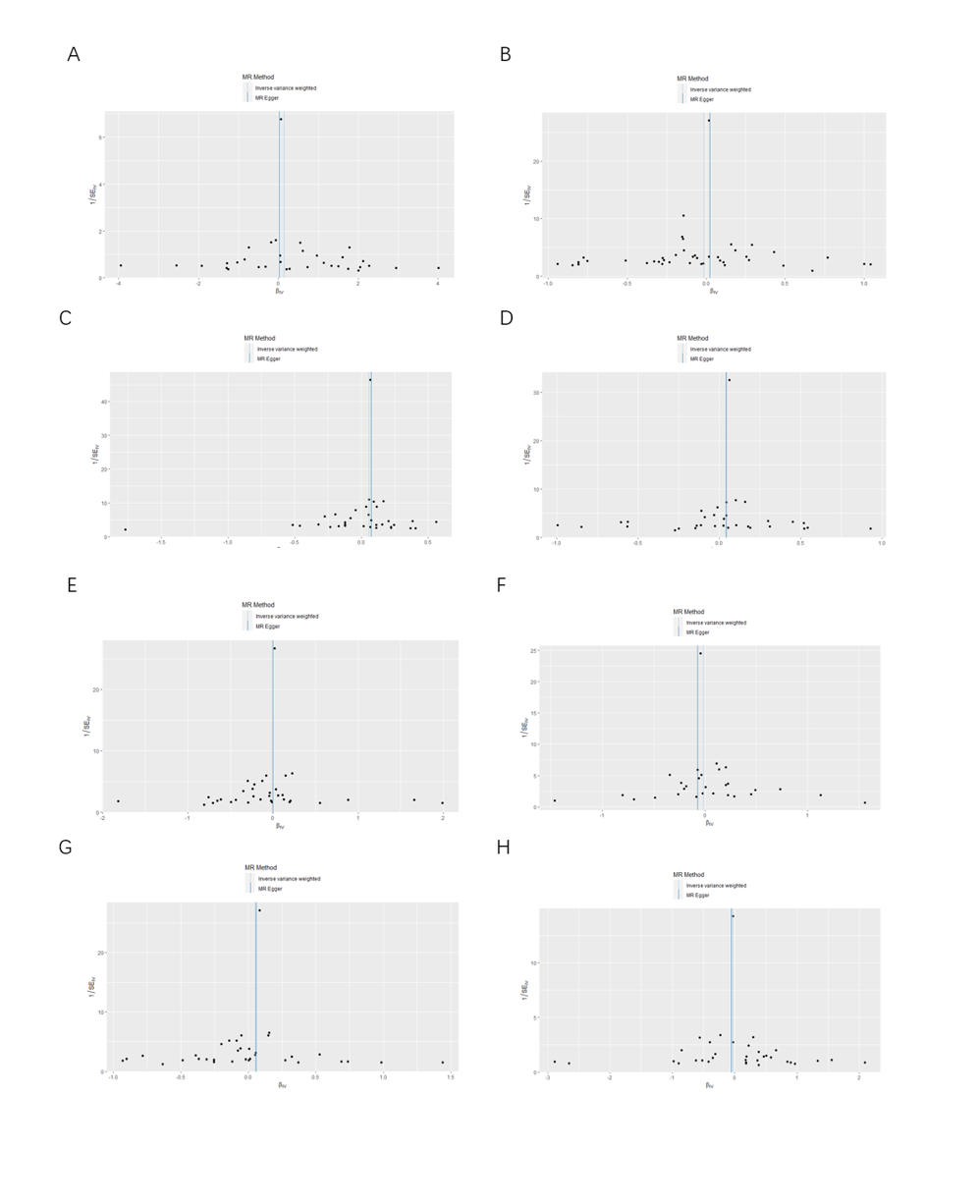

Supplement: Supplementary file 1 [file Data_Sheet_1.ZIP › Supplementary figures and tables/Supplementary Figure 15.TIFF]

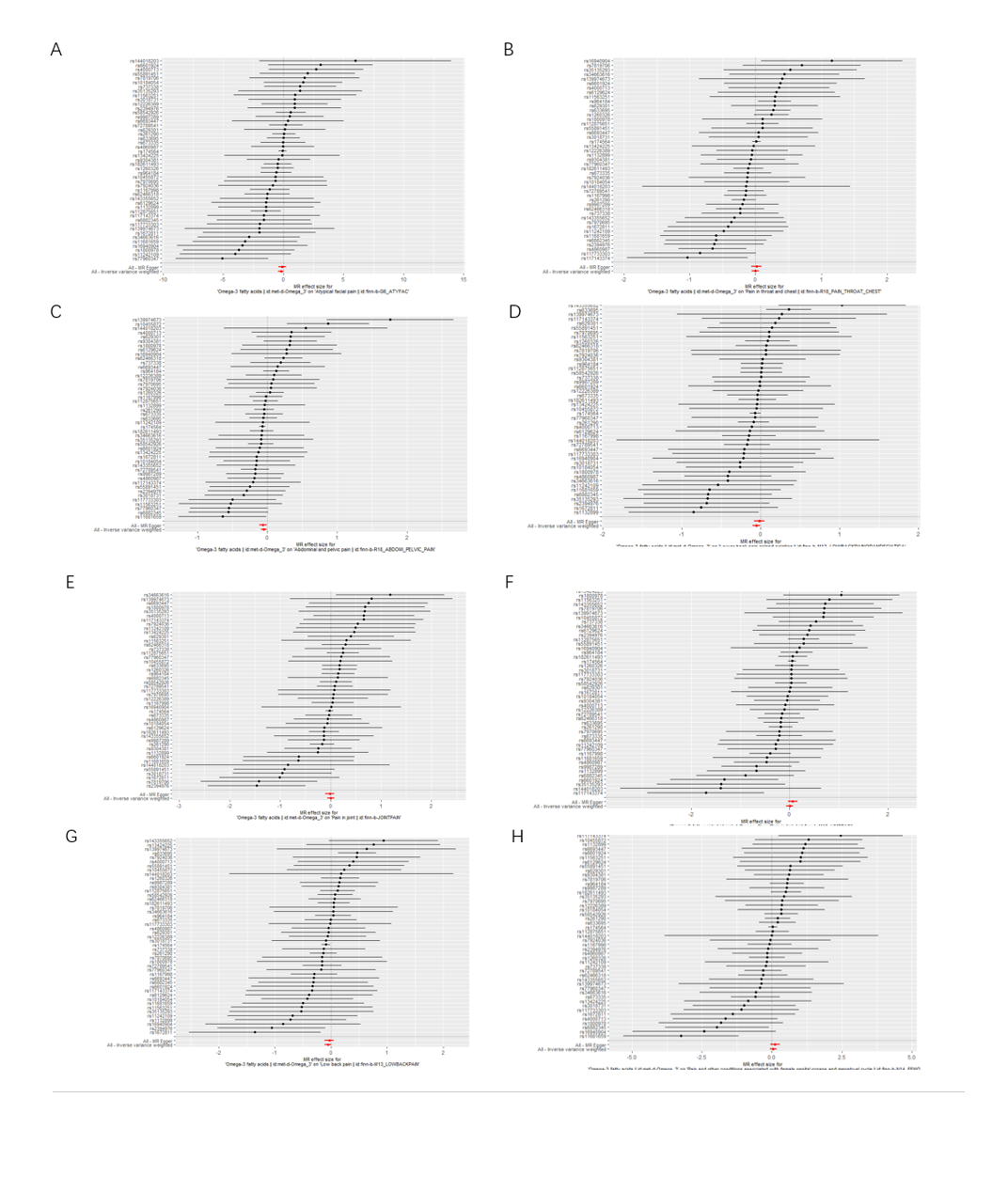

Supplement: Supplementary file 1 [file Data_Sheet_1.ZIP › Supplementary figures and tables/Supplementary Figure 16.TIFF]

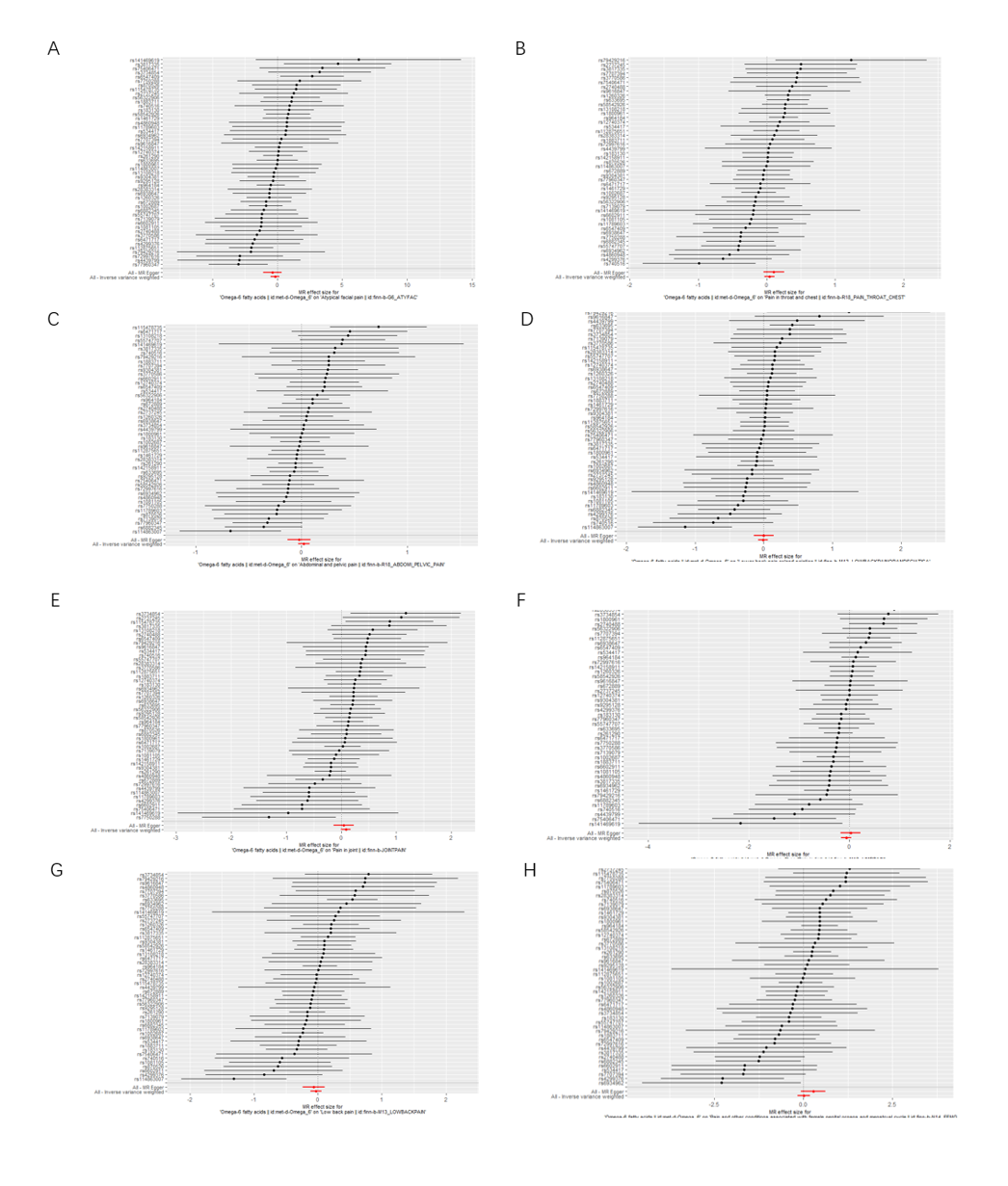

Supplement: Supplementary file 1 [file Data_Sheet_1.ZIP › Supplementary figures and tables/Supplementary Figure 17.TIFF]

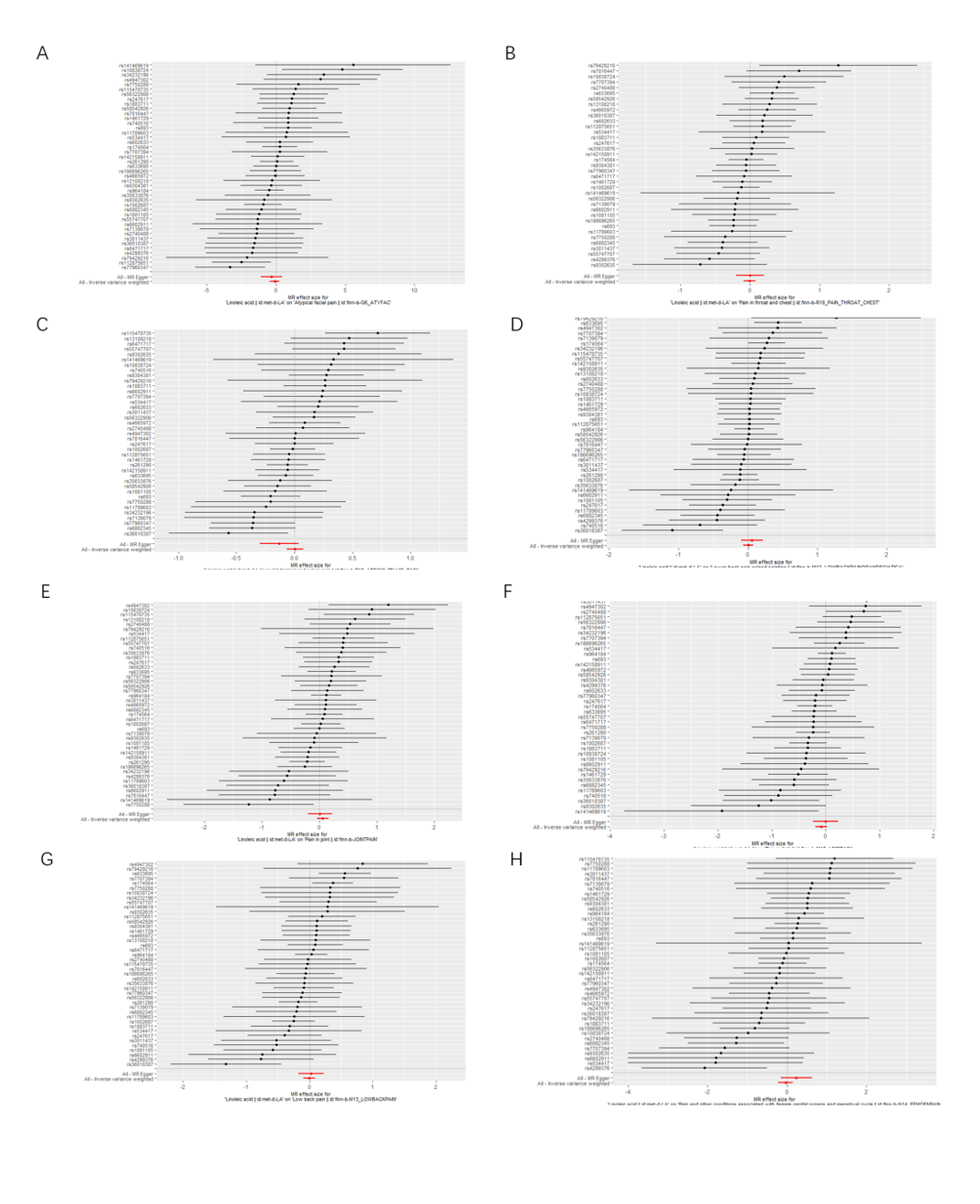

Supplement: Supplementary file 1 [file Data_Sheet_1.ZIP › Supplementary figures and tables/Supplementary Figure 18.TIFF]

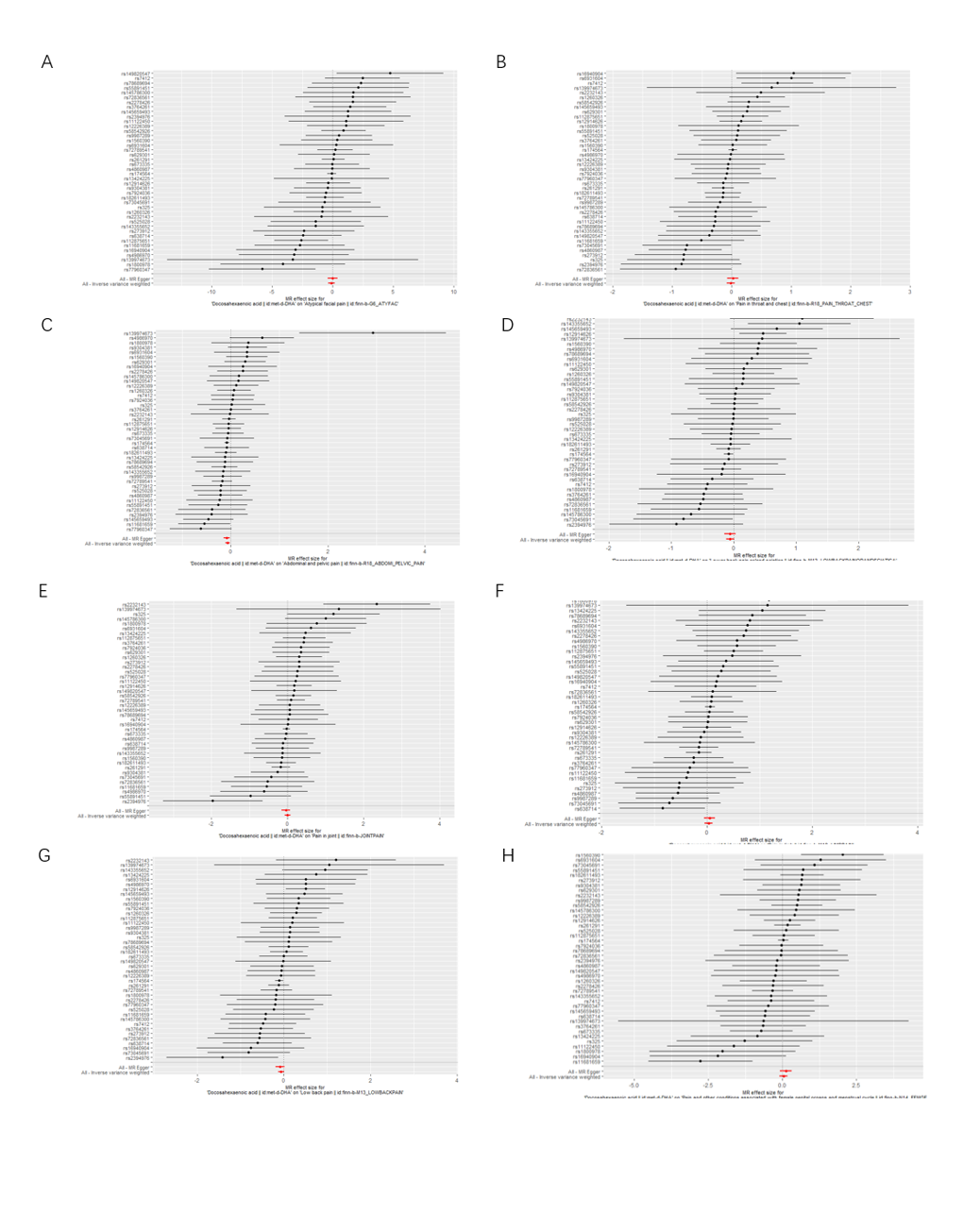

Supplement: Supplementary file 1 [file Data_Sheet_1.ZIP › Supplementary figures and tables/Supplementary Figure 19.TIFF]

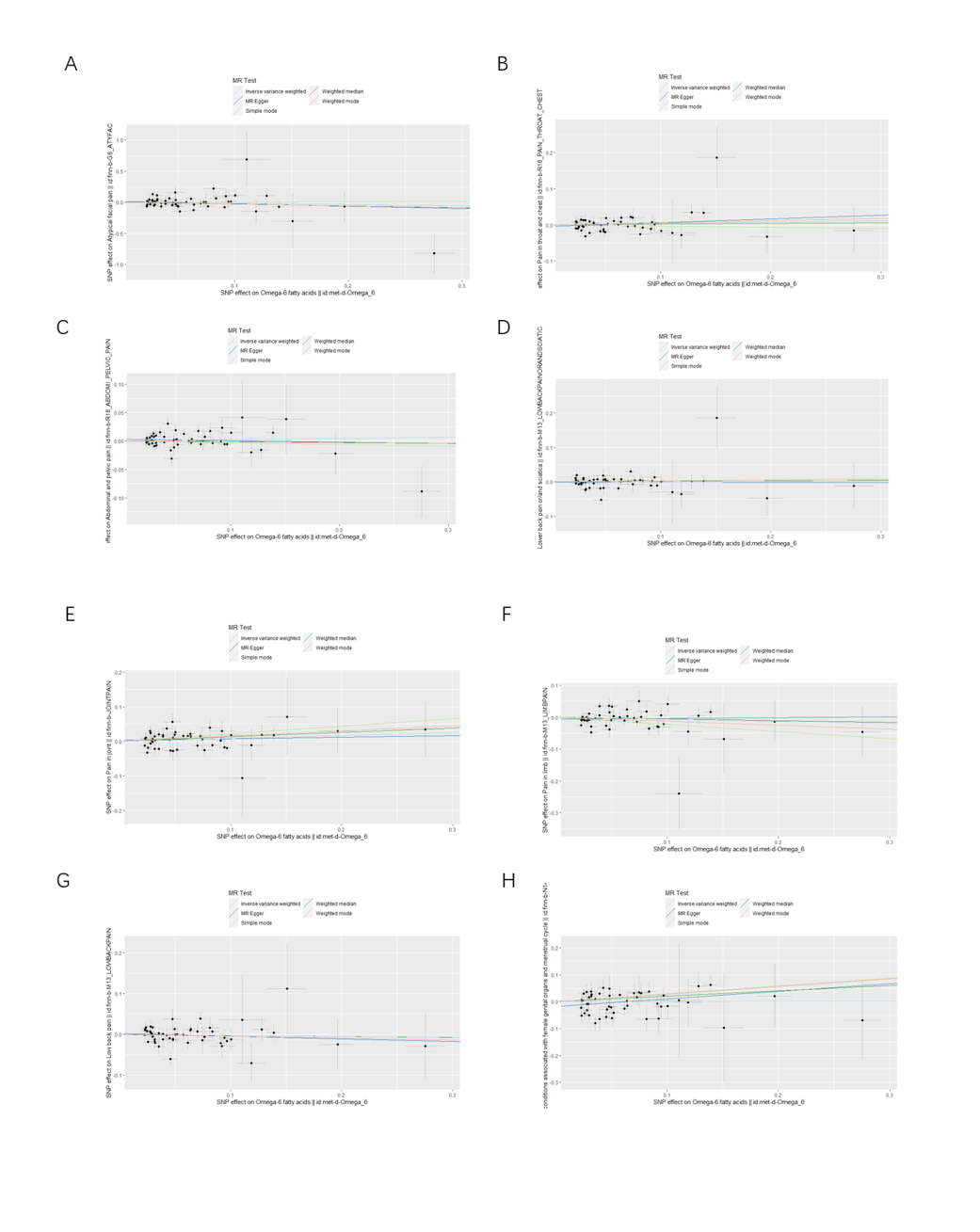

Supplement: Supplementary file 1 [file Data_Sheet_1.ZIP › Supplementary figures and tables/Supplementary Figure 2.TIFF]

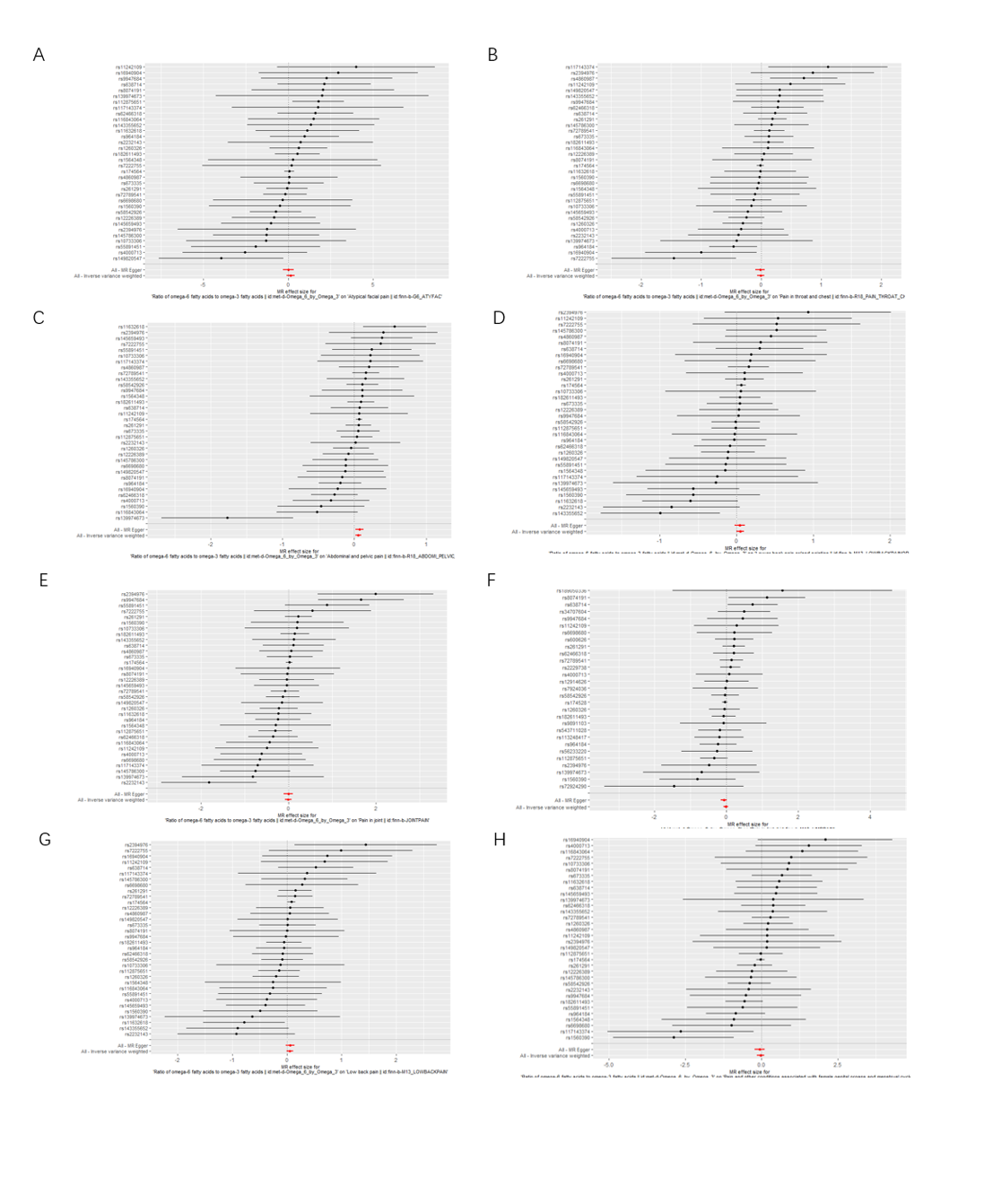

Supplement: Supplementary file 1 [file Data_Sheet_1.ZIP › Supplementary figures and tables/Supplementary Figure 20.TIFF]

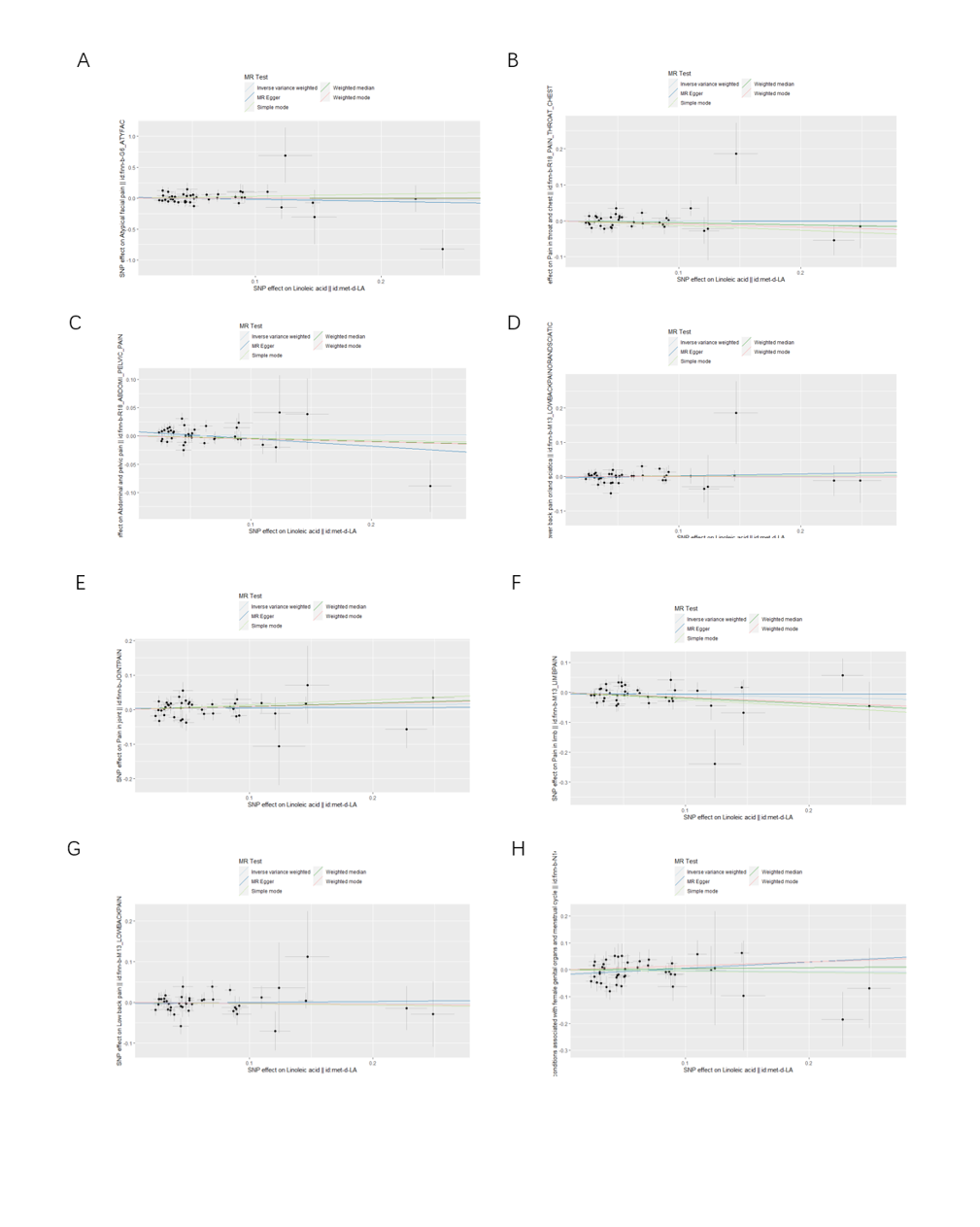

Supplement: Supplementary file 1 [file Data_Sheet_1.ZIP › Supplementary figures and tables/Supplementary Figure 3.TIFF]

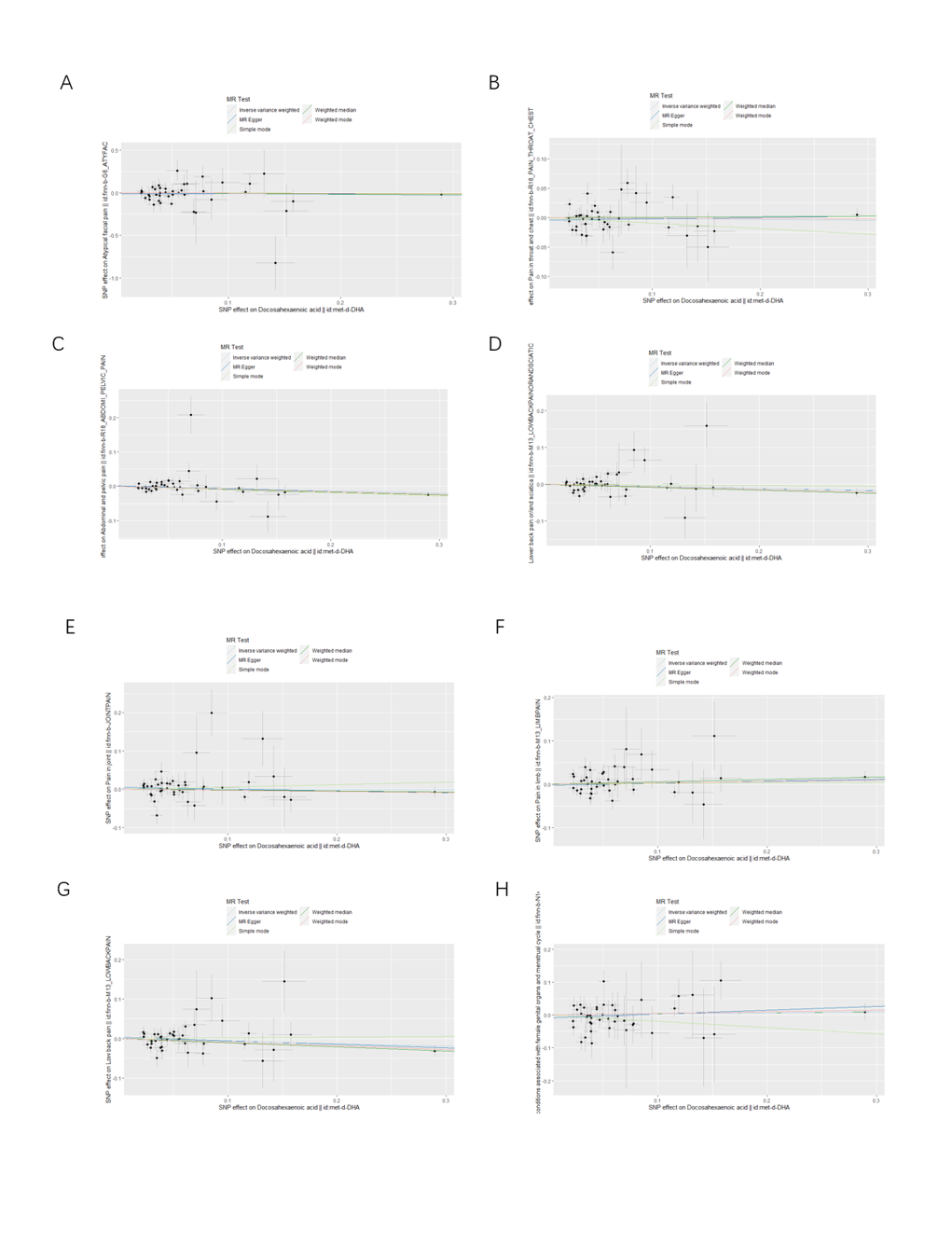

Supplement: Supplementary file 1 [file Data_Sheet_1.ZIP › Supplementary figures and tables/Supplementary Figure 4.TIFF]

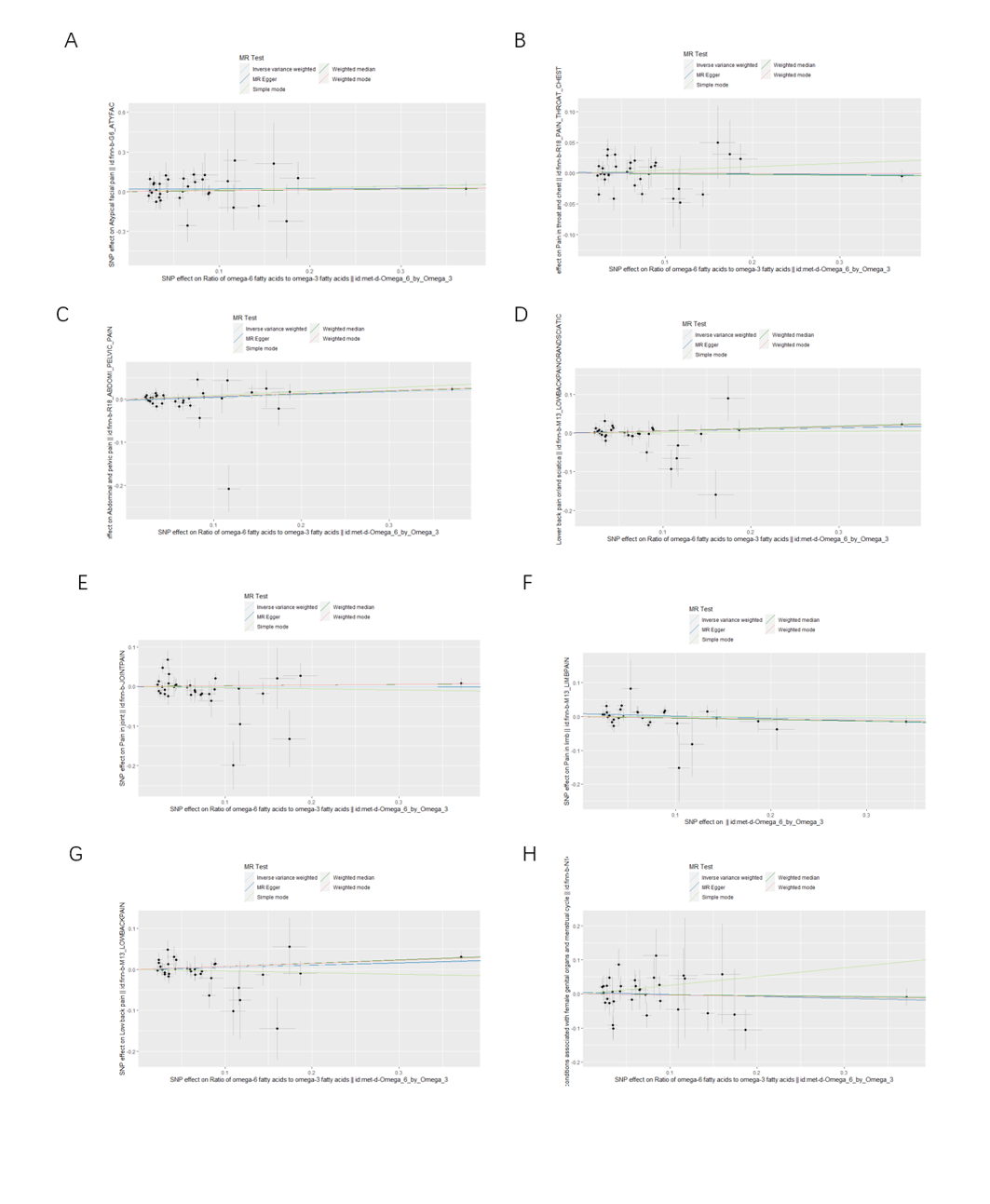

Supplement: Supplementary file 1 [file Data_Sheet_1.ZIP › Supplementary figures and tables/Supplementary Figure 5.TIFF]

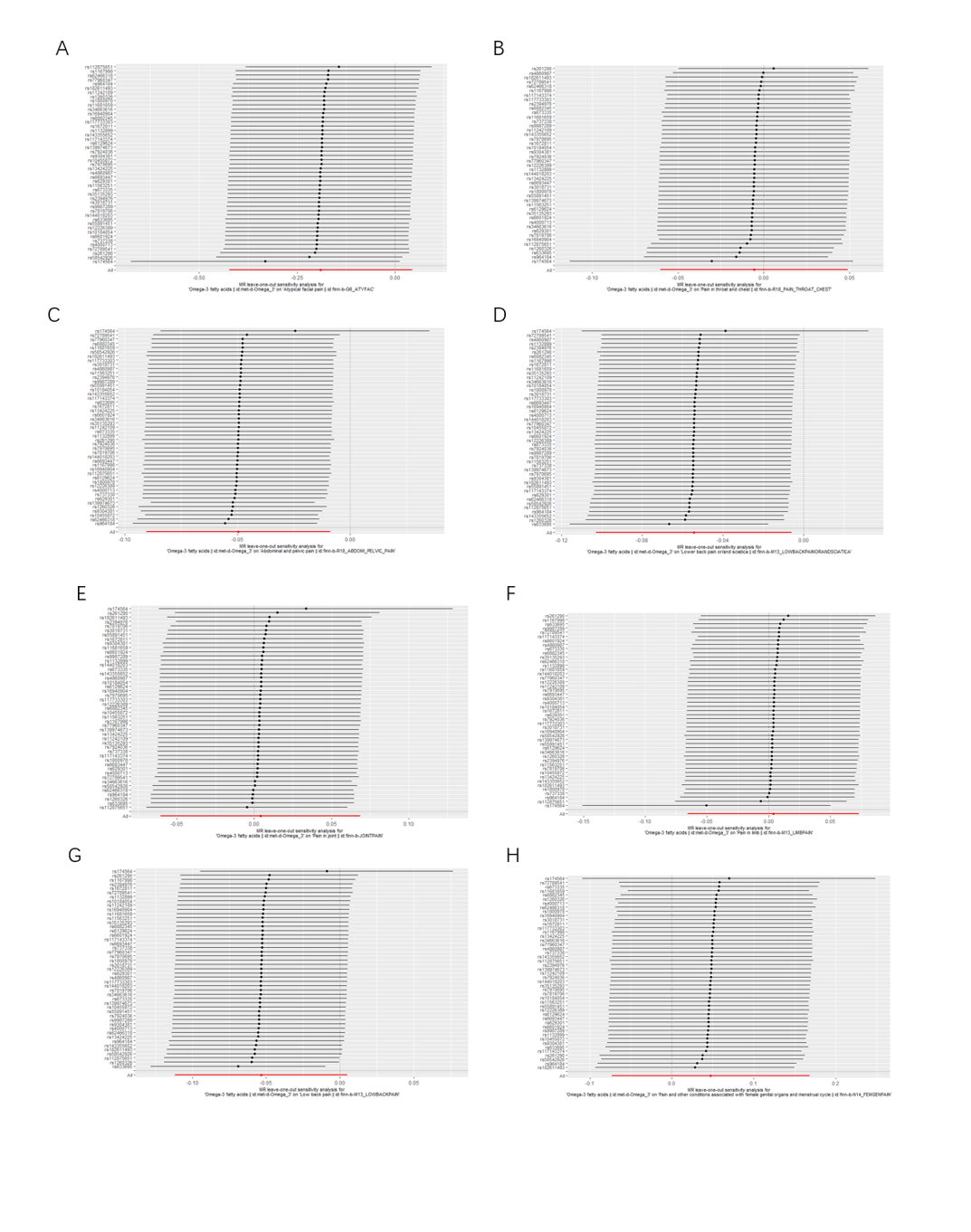

Supplement: Supplementary file 1 [file Data_Sheet_1.ZIP › Supplementary figures and tables/Supplementary Figure 6.TIFF]

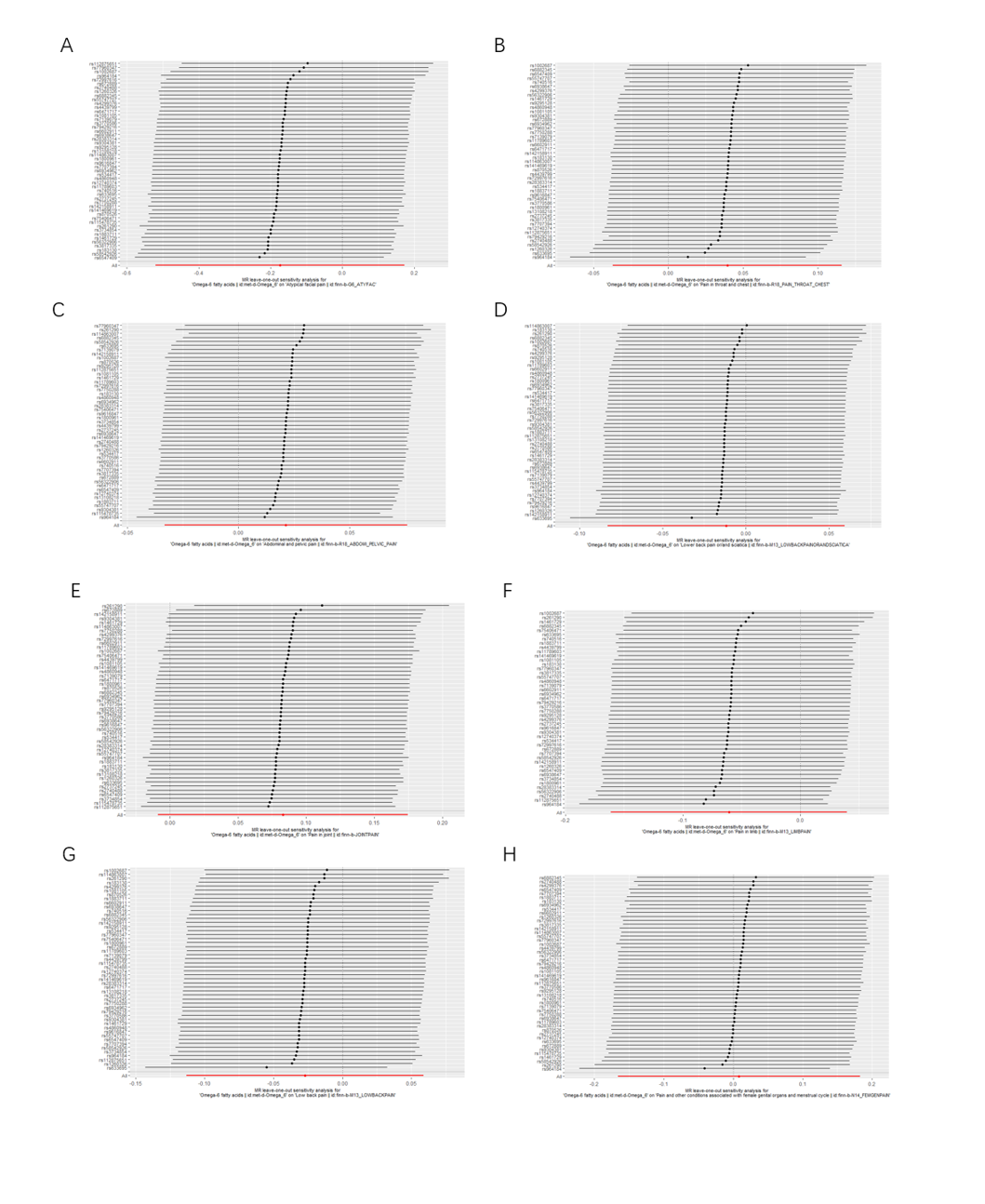

Supplement: Supplementary file 1 [file Data_Sheet_1.ZIP › Supplementary figures and tables/Supplementary Figure 7.TIFF]

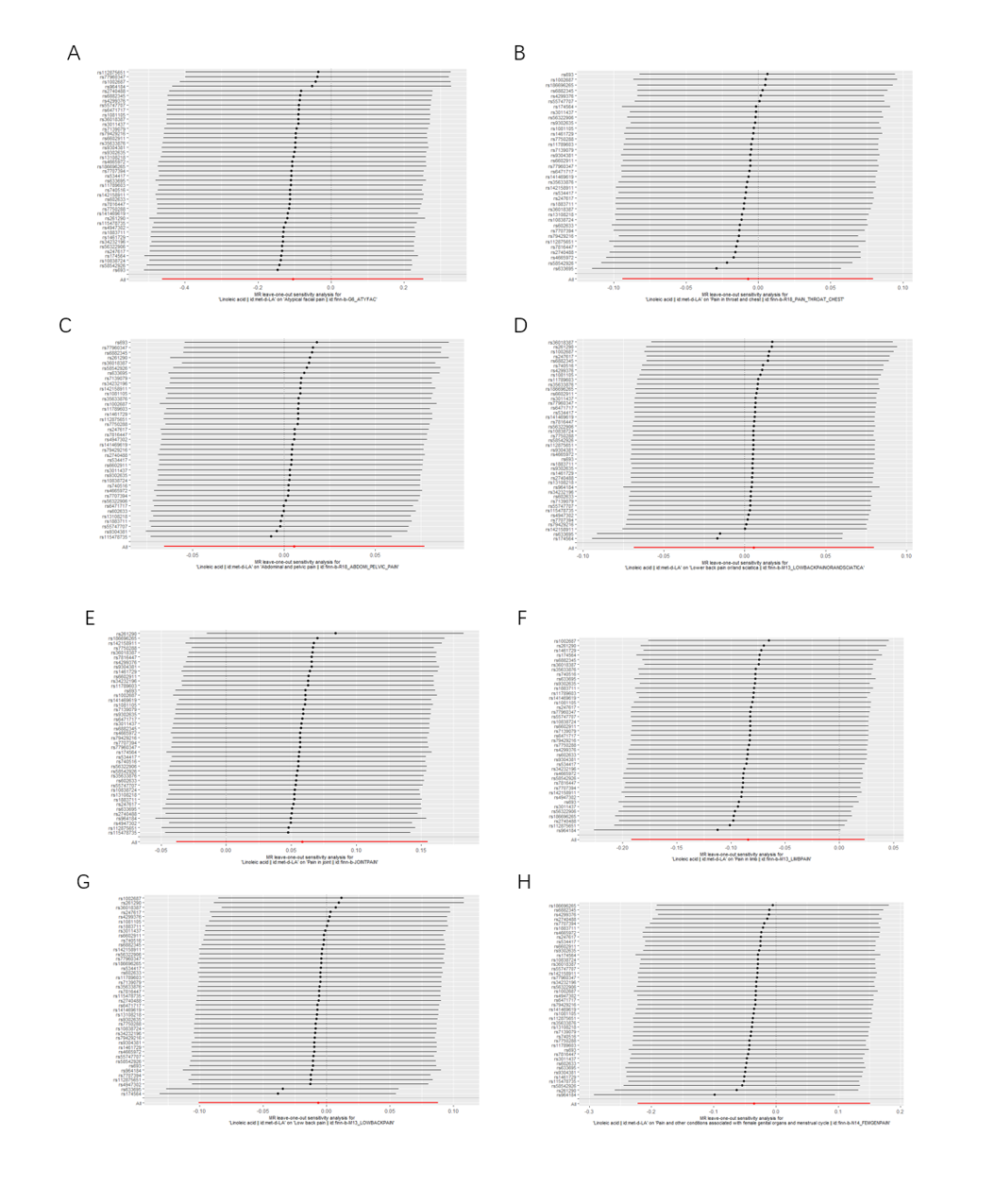

Supplement: Supplementary file 1 [file Data_Sheet_1.ZIP › Supplementary figures and tables/Supplementary Figure 8.TIFF]

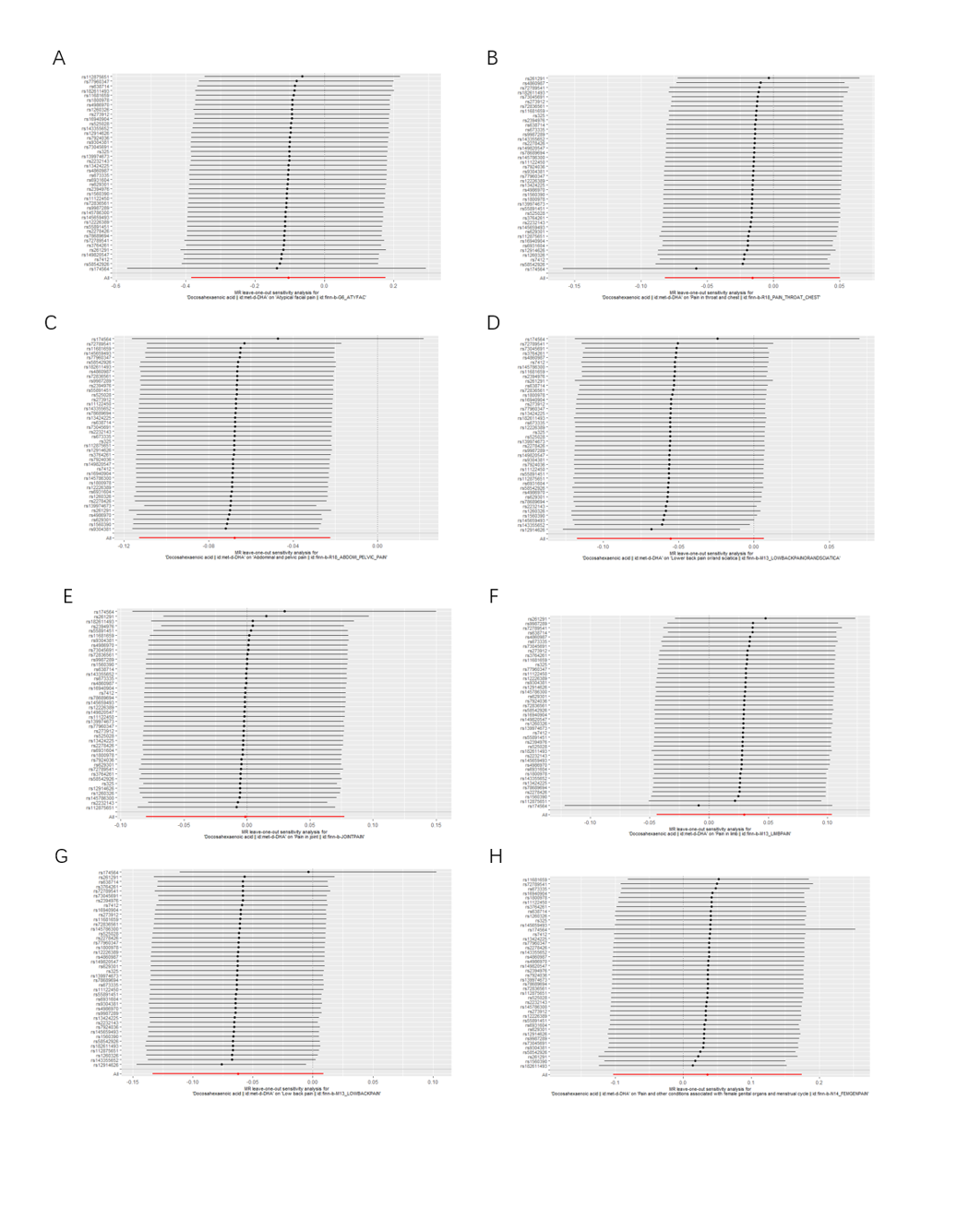

Supplement: Supplementary file 1 [file Data_Sheet_1.ZIP › Supplementary figures and tables/Supplementary Figure 9.TIFF]
